# Optogenetic activation of parvalbumin and somatostatin interneurons selectively restores theta-nested gamma oscillations and oscillation-induced spike timing-dependent long-term potentiation impaired by amyloid β oligomers

**DOI:** 10.1186/s12915-019-0732-7

**Published:** 2020-01-15

**Authors:** Kyerl Park, Jaedong Lee, Hyun Jae Jang, Blake A. Richards, Michael M. Kohl, Jeehyun Kwag

**Affiliations:** 1 Department of Brain and Cognitive Engineering, Korea University, Seoul, 02841 Republic of Korea; 2 Department of Biological Sciences, University of Toronto Scarborough, Toronto, M1C 1A4 Canada; 3 Department of Physiology, Anatomy and Genetics, University of Oxford, Oxford, OX1 3PT UK

**Keywords:** Alzheimer’s disease, Amyloid beta oligomers, Hippocampus, Optogenetics, Parvalbumin interneuron, Somatostatin interneuron, Theta-nested gamma oscillations, Spike timing-dependent long-term potentiation, Synapse-specific dysfunction

## Abstract

**Background:**

Abnormal accumulation of amyloid β_1–42_ oligomers (AβO_1–42_), a hallmark of Alzheimer’s disease, impairs hippocampal theta-nested gamma oscillations and long-term potentiation (LTP) that are believed to underlie learning and memory. Parvalbumin-positive (PV) and somatostatin-positive (SST) interneurons are critically involved in theta-nested gamma oscillogenesis and LTP induction. However, how AβO_1–42_ affects PV and SST interneuron circuits is unclear. Through optogenetic manipulation of PV and SST interneurons and computational modeling of the hippocampal neural circuits, we dissected the contributions of PV and SST interneuron circuit dysfunctions on AβO_1–42_-induced impairments of hippocampal theta-nested gamma oscillations and oscillation-induced LTP.

**Results:**

Targeted whole-cell patch-clamp recordings and optogenetic manipulations of PV and SST interneurons during in vivo-like, optogenetically induced theta-nested gamma oscillations in vitro revealed that AβO_1–42_ causes synapse-specific dysfunction in PV and SST interneurons. AβO_1–42_ selectively disrupted CA1 pyramidal cells (PC)-to-PV interneuron and PV-to-PC synapses to impair theta-nested gamma oscillogenesis. In contrast, while having no effect on PC-to-SST or SST-to-PC synapses, AβO_1–42_ selectively disrupted SST interneuron-mediated disinhibition to CA1 PC to impair theta-nested gamma oscillation-induced spike timing-dependent LTP (tLTP). Such AβO_1–42_-induced impairments of gamma oscillogenesis and oscillation-induced tLTP were fully restored by optogenetic activation of PV and SST interneurons, respectively, further supporting synapse-specific dysfunctions in PV and SST interneurons. Finally, computational modeling of hippocampal neural circuits including CA1 PC, PV, and SST interneurons confirmed the experimental observations and further revealed distinct functional roles of PV and SST interneurons in theta-nested gamma oscillations and tLTP induction.

**Conclusions:**

Our results reveal that AβO_1–42_ causes synapse-specific dysfunctions in PV and SST interneurons and that optogenetic modulations of these interneurons present potential therapeutic targets for restoring hippocampal network oscillations and synaptic plasticity impairments in Alzheimer’s disease.

## Background

Alzheimer’s disease is a neurodegenerative disease characterized by a progressive decline in cognitive and mnemonic functions [1, 2]. Abnormal accumulation of amyloid β_1–42_ oligomers (AβO_1–42_) is a hallmark of Alzheimer’s disease [1–4] and AβO_1–42_-induced impairments of gamma oscillations [5–10] and long-term synaptic plasticity [3, 4, 11, 12] are believed to contribute to the memory deficits observed in Alzheimer’s disease. In particular, hippocampal theta-nested gamma oscillations observed during spatial memory processing [13–15] have been shown to support the induction of long-term potentiation (LTP) [16–19]. Thus, AβO_1–42_ may impair memory by disrupting GABAergic inhibitory circuits, which underlie oscillogenesis [14, 20–25]. Indeed, there is now increasing experimental evidence showing that AβO_1–42_ reduces GABA synaptic transmission [26–28], causes excitation/inhibition imbalances [9, 12, 27, 28], and even diminishes the number of GABAergic synapses/terminals onto pyramidal cells [29]. Also, parvalbumin-positive (PV) and somatostatin-positive (SST) interneurons, the two major subtypes of hippocampal interneurons [30] that are critically involved in oscillogenesis [24, 25, 31], are reported to be impaired in mouse models of Alzheimer’s disease [5–8, 27, 32, 33]. PV interneurons’ spike amplitude, membrane potential, and firing rate are decreased [5, 7] while SST interneurons’ structural plasticity and axonal sprouting are impaired in Alzheimer’s disease mouse models [27, 32]. Surprisingly, the neural circuit mechanism by which dysfunction of PV and SST interneurons contributes to AβO_1–42_-induced impairment of oscillogenesis and LTP is unclear. If uncovered, it could help researchers find novel therapeutic targets for Alzheimer’s disease. Recently, optogenetic stimulation of channelrhodopsin-2 (ChR2)-expressing hippocampal CA1 pyramidal cells (PCs) at theta-frequency was shown to induce in vivo-like theta-nested gamma oscillations in the CA1 area of acute hippocampal slices in vitro [34]. This provides a novel model in which to perform targeted whole-cell patch-clamp recordings and selective optogenetic modulation of PV or SST interneuron activity during optogenetically induced theta-nested gamma oscillations and LTP induction. We have used this approach to investigate neural circuit dysfunction in hippocampal slices treated with AβO_1–42_. We found that AβO_1–42_ caused selective dysfunctions in reciprocal synapses between PC and PV interneurons, which impaired gamma oscillations and desynchronized the spike phases of PC and PV interneurons relative to gamma oscillations. While AβO_1–42_ had no effect on PC-to-SST or SST-to-PC synapses, it specifically disrupted SST interneuron-mediated disinhibition to PC resulting in the impairment of theta-nested gamma oscillation-induced spike timing-dependent LTP (tLTP). Selective optogenetic activation of PV interneurons restored gamma oscillations while selective optogenetic activation of SST interneurons restored theta-nested gamma oscillation-induced tLTP. These results demonstrate that AβO_1–42_-induced synapse-specific dysfunctions in PV and SST interneurons may explain the concomitant impairments of hippocampal gamma oscillations and synaptic plasticity in Alzheimer’s disease. Moreover, using a computational network model of PC, PV, and SST interneurons, we further demonstrate that PV and SST interneurons targeting different compartments of the CA1 PC have distinct functional roles in oscillogenesis and tLTP induction.

## Results

### AβO_1–42_ impairs in vivo-like, optogenetically induced theta-nested gamma oscillations in hippocampal slices

**Fig. 1:**
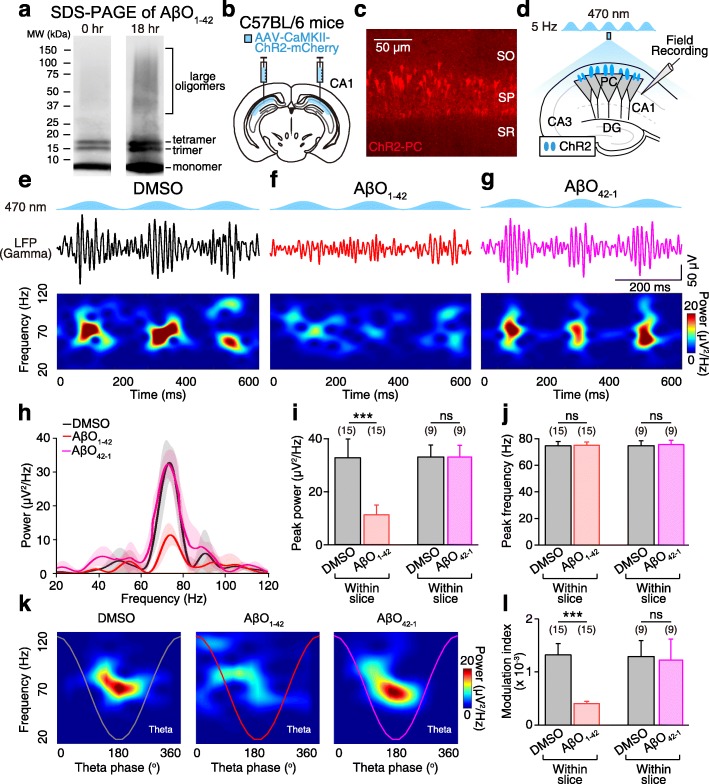
AβO_1–42_ impairs in vivo-like, optogenetically induced theta-nested gamma oscillations in hippocampal slices*.* **a** Western blot of SDS-PAGE showing AβO_1–42_ (trimer, tetramer, and large oligomers) after incubation at 4 °C for 0 h (left) and 18 h (right). **b** Micro-injection of AAV-CaMKII-ChR2-mCherry into hippocampal CA1 area of C57BL/6 mice. **c** Fluorescence image of ChR2-expressing PCs (ChR2-PC). SO, stratum oriens; SP, stratum pyramidale; SR, stratum radiatum. **d** Experimental schematic showing sinusoidal (5 Hz) blue light (470 nm) stimulation of ChR2-PC and field recordings in the CA1 area of hippocampal slices in vitro. **e–g** Sinusoidal blue light stimulation induces theta-nested gamma oscillations as shown in the band-pass filtered LFP (top) and the corresponding spectrograms (bottom) in DMSO-treated slice (**e**), after 20-min treatment of either AβO_1–42_ (**f**), or AβO_42–1_ (**g**). **h–j** Mean power spectral density (PSD, shade indicates SEM) of gamma oscillations (**h**), mean peak power (**i**), and mean peak frequency (**j**) of gamma oscillations in DMSO-treated slice (black) and following 20 min of AβO_1–42_ treatment in the same slices (red) or in DMSO-treated slice (black) and following 20 min AβO_42–1_ treatment in the same slices (magenta). **k**, **l** Representative comodulograms showing phase-amplitude coupling of gamma oscillations to theta cycle (**k**) and mean modulation index (**l**) in each condition. Paired Student’s *t* test (**i**, **j**, **l**, ***p < 0.001, ns: not significant). Data are represented as mean ± SEM

To create an in vitro model of AβO_1–42_-induced pathology in hippocampal slices, we prepared AβO_1–42_ by oligomerizing Aβ_1–42_ following a previously described protocol [4] (see the “Methods” section). Generation of AβO_1–42_ was confirmed by Western blot analysis of SDS-PAGE (Fig. 1a) and native PAGE (Additional file 1: Figure S1). To induce blue light-induced theta-nested gamma oscillations, we injected adeno-associated virus (AAV) carrying ChR2 (AAV-CaMKII-ChR2-mCherry) into the CA1 area of the hippocampus (Fig. 1b), which led to the expression of ChR2 in CA1 PCs in hippocampal slices in vitro (Fig. 1c). We optically stimulated ChR2-expressing PCs using 5 Hz sinusoidal blue light (470 nm, Fig. 1d) in dimethyl sulfoxide (DMSO)-treated hippocampal slices which reliably reproduced theta-nested gamma oscillations as observed in the band-pass filtered local field potential (LFP) (Fig. 1e, black traces, top) and in the spectrogram [34] (Fig. 1e, bottom) that persisted for over 40 min (Additional file 2: Figure S2). However, 20-min treatment of AβO_1–42_ (200 nM) in the same slice significantly decreased the power of gamma oscillations in the LFP (Fig. 1f, red traces, top) and in the spectrogram (Fig. 1f, bottom), while 20-min treatment of AβO_42–1_, an inactive peptide control for AβO_1–42_, in the same slice of DMSO-treated slices had no effect (Fig. 1g, magenta). Power spectral density (PSD) analysis of theta-nested gamma oscillations (Fig. 1h) revealed that peak power of gamma oscillations in the DMSO-treated slice (Fig. 1i, black) was impaired by AβO_1–42_ (Fig. 1i, red), but not by AβO_42–1_ (Fig. 1i, magenta), while peak frequency was spared in all conditions (Fig. 1j). Moreover, phase-amplitude coupling analysis of gamma oscillations to the trough of theta cycle (Fig. 1k) revealed that the coupling strength, quantified by the modulation index (see the “Methods” section), was significantly decreased by AβO_1–42_, but not by AβO_42–1_, compared to that in the DMSO-treated slices (Fig. 1l). We replicated these effects in different slices treated with AβO_1–42_ for 20 min before performing field recording (Additional file 3: Figure S3); thus, the reduction in oscillatory activity was not caused by recording duration. These results show that AβO_1–42_-treated slices with optical stimulation of ChR2-expressing CA1 PCs can replicate gamma oscillations impairment as observed in Alzheimer’s disease mouse models in vivo [5–8].

### AβO_1–42_ causes synapse-specific dysfunction of PC-to-PV, but not PC-to-SST synapses

**Fig. 2:**
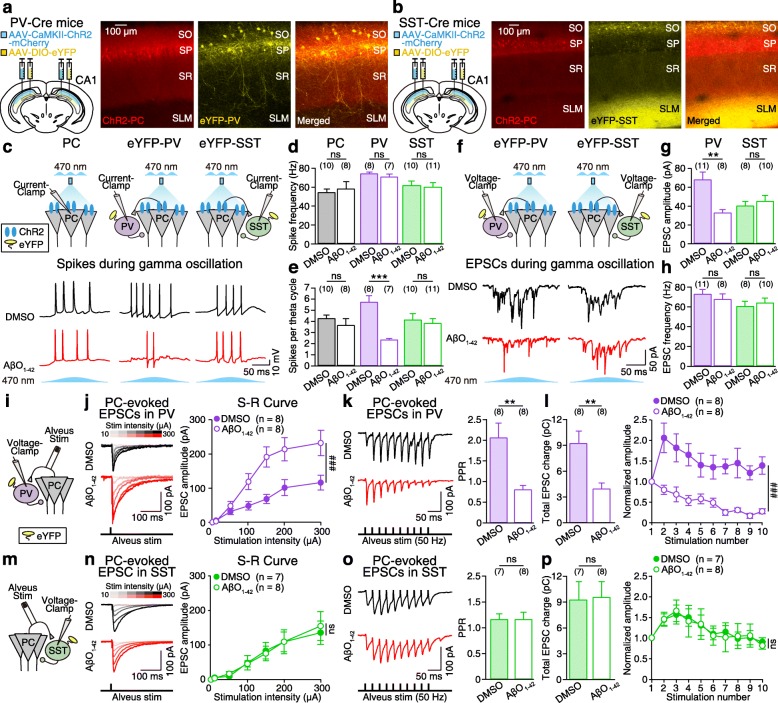
AβO_1–42_ causes synapse-specific dysfunction of PC-to-PV, but not PC-to-SST synapses. **a**, **b** Micro-injection of AAV-CaMKII-ChR2-mCherry and AAV-DIO-eYFP into CA1 area (left) and fluorescence image (right) of ChR2-expressing PCs (ChR2-PC) with eYFP-expressing PV interneurons (eYFP-PV) in PV-Cre mice (**a**) and ChR2-PC with eYFP-expressing SST interneurons (eYFP-SST) in SST-Cre mice (**b**). SO, stratum oriens; SP, stratum pyramidale; SR, stratum radiatum; SLM, stratum lacunosum-moleculare. **c** Experimental schematic. Whole-cell current-clamp recordings in CA1 PC, eYFP-PV, or eYFP-SST during sinusoidal (5 Hz) blue light (470 nm) stimulation (top) and representative spikes (bottom) in DMSO-treated (black) and AβO_1–42_-treated slices (red). **d**, **e** Mean spike frequency (**d**) and the number of spikes per theta cycle (**e**) recorded in CA1 PC (black), eYFP-PV (purple), and eYFP-SST (green). **f** Experimental schematic. Whole-cell voltage-clamp recordings in eYFP-PV/eYFP-SST during sinusoidal blue light stimulation (top) and representative EPSCs (bottom) in DMSO-treated (black) and AβO_1–42_-treated slices (red). **g**, **h** Mean EPSC amplitude (**g**) and mean EPSC frequency (**h**) in eYFP-PV (purple) and eYFP-SST (green). **i** Experimental schematic. Alveus stimulation to record PC-evoked EPSCs in eYFP-PV. **j** Representative PC-evoked EPSCs from eYFP-PV (left) and stimulus-response (S-R) curve (right) in DMSO-treated and AβO_1–42_-treated slices. **k**, **l** Representative PC-evoked EPSCs from eYFP-PV in response to alveus stimulation (10 pulses, 50 Hz, **k**, left), paired-pulse ratio (PPR) of the 2nd EPSC/1st EPSC (**k**, right), total EPSC charge (**l**, left), and EPSCs normalized to the 1st EPSC to show short-term plasticity (**l**, right) in DMSO-treated (filled circles) and AβO_1–42_-treated slices (empty circles). **m–p** Same as **i–l** but with PC-evoked EPSCs in eYFP-SST. Unpaired Student’s *t* test (**d**, **e**, **g**, **h**, **k**, **l** (left), **o**, **p** (left), ***p < 0.001, **p < 0.01, ns: not significant), two-way ANOVA with post hoc Tukey’s test (**j**, **l** (right), **n**, **p** (right), ^###^*p* < 0.001, ns: not significant). Data are represented as mean ± SEM

To determine whether alterations to either PV or SST interneurons contributed to the reduction in peak power of gamma oscillations in the AβO_1–42_-treated slices, we expressed ChR2 in CA1 PCs and enhanced yellow fluorescent protein (eYFP) in either PV or SST interneurons in PV-Cre (Fig. 2a) or SST-Cre mice (Fig. 2b), respectively. We then performed current-clamp recordings to record spikes in CA1 PCs, eYFP-expressing PV, and SST interneurons during blue light-induced theta-nested gamma oscillations (Fig. 2c). We found that all neuronal types spiked at gamma-frequency in DMSO-treated slices (Fig. 2c, black traces, Fig. 2d). AβO_1–42_ had no effect on neither spike frequencies (Fig. 2c, red traces, Fig. 2d), nor the intrinsic membrane properties (Additional file 4: Figure S4) of PV and SST interneurons, which could explain why the peak frequency of gamma oscillations was intact even after AβO_1–42_ treatment (Fig. 1j). However, the number of spikes per theta cycle was reduced only in PV interneurons (Fig. 2e).

Since the spiking of hippocampal CA1 interneurons is in large part driven by CA1 PC’s excitatory inputs to the interneurons [35], we investigated whether the treatment of AβO_1–42_ affected CA1 PC’s excitatory inputs to PV and SST interneurons. We performed voltage-clamp recordings in eYFP-expressing PV or SST interneurons during blue light-induced theta-nested gamma oscillations in DMSO-treated and AβO_1–42_-treated slices (Fig. 2f). We found that the amplitude of CA1 PC’s excitatory postsynaptic current (EPSC) to PV, but not SST interneuron, was significantly decreased in AβO_1–42_-treated slices (Fig. 2f, g), while EPSC frequency was unaffected (Fig. 2h). To characterize the AβO_1–42_-induced synaptic dysfunctions at CA1 PC-to-PV synapse and CA1 PC-to-SST synapse, we first investigated how AβO_1–42_ affected the stimulus-response (S-R) curve of these synapses by electrically stimulating the axons of CA1 PC in the alveus of CA1 at different intensities (10, 50, 100, 150, 200, and 300 μA) and recording the corresponding PC-evoked EPSCs in eYFP-expressing PV interneuron (Fig. 2i, j) or in eYFP-expressing SST interneuron (Fig. 2m, n). Analysis of the S-R curve revealed that, for each stimulation intensity, AβO_1–42_ significantly increased the amplitudes of PC-evoked EPSCs in PV (Fig. 2j, right), but not those in SST interneurons (Fig. 2n, right). These results indicate that AβO_1–42_ increases the initial neurotransmitter release probability of PC-to-PV synapse. To investigate the synaptic locus of EPSC changes, we stimulated the CA1 PC axons using a half-maximal stimulus (based on the S-R curve in Fig. 2j, n, right; 115–210 μA) and an inter-stimulus interval of 20 ms (50 Hz, 10 stimulus) for the analysis of paired-pulse ratio (PPR), total charge, and short-term plasticity of PC-evoked EPSCs in PV (Fig. 2k, l) and SST interneurons (Fig. 2o, p). Paired-pulse facilitation of PC-evoked EPSCs in PV interneurons, as observed in DMSO-treated slices, was converted to paired-pulse depression in AβO_1–42_-treated slices (Fig. 2k, right). The total charge of PC-evoked EPSCs in PV (Fig. 2l, left), analyzed by the area of the PC-evoked EPSCs in Fig. 2k (left), was significantly decreased by AβO_1–42_. Furthermore, short-term facilitation of PC-evoked EPSCs in PV interneurons, as observed in DMSO-treated slices, was converted to short-term depression in AβO_1–42_-treated slices (Fig. 2l, right). These results indicate that AβO_1–42_ causes presynaptic depression at PC-to-PV synapse, which led to a decrease in CA1 PC-evoked excitatory synaptic inputs onto PV interneurons. Thus, AβO_1–42_-induced gamma oscillation impairment may be due to dysfunction of presynaptic mechanisms at PC-to-PV synapses. In contrast, AβO_1–42_ had no effect on PPR, total charge, or short-term plasticity of CA1 PC-evoked EPSCs in SST interneurons (Fig. 2o, p). Therefore, AβO_1–42_ causes presynaptic dysfunctions at CA1 PC-to-interneuron synapses which is target-specific.

### AβO_1–42_ causes synapse-specific dysfunction of PV-to-PC synapses, but not SST-to-PC synapses

**Fig. 3:**
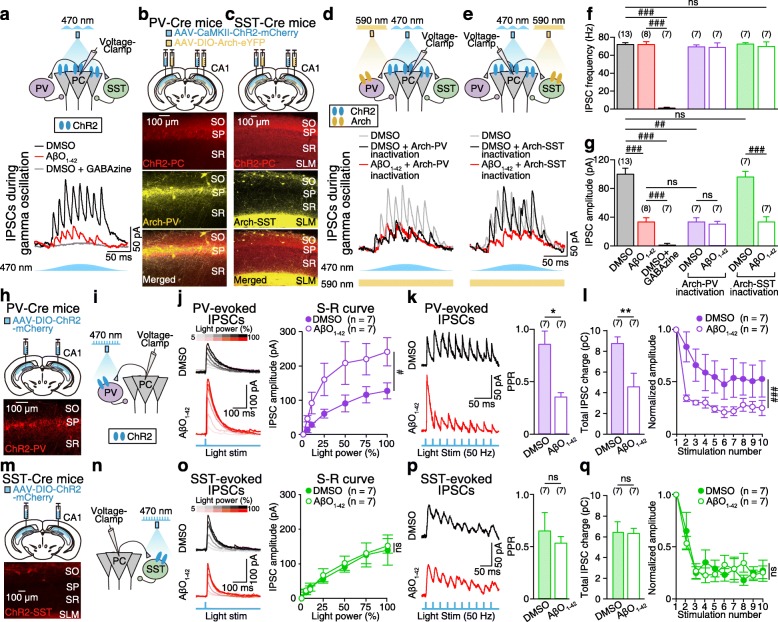
AβO_1–42_ causes synapse-specific dysfunction of PV-to-PC synapses, but not SST-to-PC synapses. **a** Experimental schematic. Whole-cell voltage-clamp recordings in CA1 PC (top) and representative IPSCs (bottom) during blue light-induced gamma oscillations in DMSO-treated (black), AβO_1–42_-treated slices (red), and DMSO-treated slice with GABAzine (gray). **b**, **c** Micro-injection of AAV-CaMKII-ChR2-mCherry and AAV-DIO-Arch-eYFP into CA1 area (top) and fluorescence image (bottom) of ChR2-expressing PCs (ChR2-PC) with Arch-expressing PV interneurons (Arch-PV) in PV-Cre mice (**b**) and ChR2-PC with Arch-expressing SST interneurons (Arch-SST) in SST-Cre mice (**c**). **d**, **e** Same as **a** but with inactivation of Arch-PV (**d**) and Arch-SST (**e**) using tonic yellow light (590 nm) stimulation in DMSO- and AβO_1–42_-treated slice. **f**, **g** Mean IPSC frequency (**f**) and mean IPSC amplitude (**g**) in each condition. **h** Micro-injection of AAV-DIO-ChR2-mCherry into CA1 area of PV-Cre mice (top) and fluorescence image (bottom) of ChR2-expressing PV interneurons (ChR2-PV). **i**, **j** Experimental schematic. Whole-cell voltage-clamp recordings in CA1 PC (**i**) to record PV-evoked IPSCs (**j**, left) and stimulus-response (S-R) curve (**j**, right) in response to different light stimulation powers. **k**, **l** Representative PV-evoked IPSCs in CA1 PC in response to light stimulation (10 pulses, 50 Hz, **k**, left), paired-pulse ratio (PPR) of the 2nd IPSC/1st IPSC (**k**, right), total IPSC charge (**l**, left), and IPSCs normalized to the 1st IPSC to show short-term plasticity (**l**, right) in DMSO-treated (filled circles) and AβO_1–42_-treated slices (empty circles). **m–q** Same as **h–l** but by activating ChR2-expressing SST interneurons (ChR2-SST) for SST-evoked IPSCs in SST-Cre mice. Unpaired Student’s *t* test (**k**, **l** (left), **p**, **q** (left), **p < 0.01, *p < 0.05, ns: not significant), one-way (**f**, **g**, ^###^*p* < 0.001, ^##^*p* < 0.01, ns: not significant) and two-way ANOVA with post hoc Tukey’s test (**j**, **l** (right), **o**, **q** (right), ^###^*p* < 0.001, ^#^*p* < 0.05, ns: not significant). Data are represented as mean ± SEM

Blue light-induced theta-nested gamma oscillations are most likely generated by reciprocal synapses between PCs and interneurons [34], according to the pyramidal-interneuron network gamma (PING) model [14, 21, 23]. In accordance with this model, voltage-clamp recordings in CA1 PCs during blue light-induced gamma oscillations (Fig. 3a, top) revealed that inhibitory postsynaptic currents (IPSCs) occurred at gamma-frequencies in DMSO-treated slices (Fig. 3a, bottom, black trace, Fig. 3f), which were GABA_A_ receptor-mediated as they were completely blocked by GABAzine (SR95531, 5 μM, Fig. 3a, bottom, gray trace; Fig. 3f, g). AβO_1–42_ significantly decreased the amplitude of these IPSCs (Fig. 3a, bottom, red trace; Fig. 3g), potentially accounting for the observed reduction in peak power of gamma in AβO_1–42_-treated slices (Fig. 1h, i). To determine which interneuron subtype was responsible for the reduction of IPSC in PC in AβO_1–42_-treated slices, we optogenetically inactivated either PV or SST interneuron during gamma oscillations by co-injecting two different AAV viruses to CA1, one carrying ChR2 and the other carrying enhanced Arch (AAV-DIO-Arch-eYFP) in order to express ChR2 in PCs and Arch in either PV (Fig. 3b) or SST interneurons (Fig. 3c). During theta-nested gamma oscillations in DMSO-treated slices, inactivation of Arch-expressing PV interneurons (Fig. 3d) and Arch-expressing SST interneurons (Fig. 3e) by yellow light (590 nm) had no effect on IPSC frequency in CA1 PCs (Fig. 3f). However, IPSC amplitude in CA1 PC was significantly reduced only by inactivation of Arch-expressing PV interneurons in the DMSO-treated slices (Fig. 3g), which was similar to that recorded in AβO_1–42_-treated slices (Fig. 3a, red trace, Fig. 3g). Inactivation of Arch-expressing PV interneurons in AβO_1–42_-treated and DMSO-treated slices had the same effect in reducing IPSC amplitudes (Fig. 3d, red trace, Fig. 3g) while inactivation of Arch-expressing SST interneurons in AβO_1–42_-treated slices significantly reduced the IPSC amplitude compared to that in the DMSO-treated slices (Fig. 3e, red traces, Fig. 3g). Moreover, the peak power of gamma oscillations was also decreased only by inactivation of Arch-expressing PV interneuron (Additional file 5: Figure S5) while inactivation of Arch-expressing SST interneuron had no effect on gamma oscillations (Additional file 6: Figure S6), indicating AβO_1–42_-induced reduction of IPSC in CA1 PCs as well as the reduction of peak power of gamma oscillations may be due to dysfunction of PV interneurons. To rule out the possibility of yellow light having any direct effects on the reduction of gamma oscillation power via activation of ChR2 in CA1 PCs, we recorded synaptic currents in ChR2-expressing PCs and LFPs in the nearby tissue during sinusoidal (5 Hz) blue (470 nm), green (565 nm), and yellow light (590 nm) stimulation (Additional file 7: Figure S7a-c). We found that green light induced synaptic currents and gamma oscillations in the LFP while yellow light stimulation had no effect on either of them (Additional file 7: Figure S7d, e). In order to characterize the AβO_1–42_-induced synaptic dysfunctions at PV-to-CA1 PC synapse and SST-to-CA1 PC synapse, we expressed ChR2 in PV (Fig. 3h) and SST interneurons (Fig. 3m) and analyzed the S-R curve of these synapses by optically stimulating ChR2-expressing PV interneurons (Fig. 3i) and ChR2-expressing SST interneurons (Fig. 3n) at different light powers (5, 10, 25, 50, 75, 100% of maximal light power (15 mW)) and recorded the corresponding PV-evoked IPSCs in PC (Fig. 3j) and SST-evoked IPSCs in PC (Fig. 3o). Analysis of the S-R curve revealed that, for each stimulation intensity, AβO_1–42_ significantly increased the amplitudes of PV-evoked IPSCs in PC (Fig. 3j), but not SST-evoked IPSCs in PC (Fig. 3o), suggesting that AβO_1–42_ increases the initial neurotransmitter release probability of PV-to-PC synapse. To investigate the synaptic locus of IPSC changes, we optically stimulated ChR2-expressing PV interneurons and ChR2-expressing SST interneurons using a half-maximal light power (based on S-R curve in Fig. 3j, o; 3.75–9 mW) and an inter-stimulus interval of 20 ms (50 Hz, 10 stimulus) for the analysis of PPR, total charge, and short-term plasticity of PV-evoked IPSCs (Fig. 3k, l) and SST-evoked IPSCs (Fig. 3p, q). AβO_1–42_ significantly enhanced the paired-pulse depression in PV-evoked IPSCs in PC, as observed in DMSO-treated slice (Fig. 3k, right). The total charge of PV-evoked IPSCs in PC was significantly decreased by AβO_1–42_ (Fig. 3l, left). Furthermore, short-term depression of PV-evoked IPSCs in PC, as observed in DMSO-treated slice was even more enhanced in AβO_1–42_-treated slices (Fig. 3l, right) while it had no effect on SST-evoked IPSCs (Fig. 3p, q). Together, these results indicate that AβO_1–42_ specifically disrupted reciprocal PC-to-PV and PV-to-PC synapses, which would likely impair gamma oscillations, while AβO_1–42_ had no effect on PC-to-SST or SST-to-PC synapses.

### Optogenetic activation of PV interneurons restores AβO_1–42_-induced impairment of theta-nested gamma oscillations

**Fig. 4:**
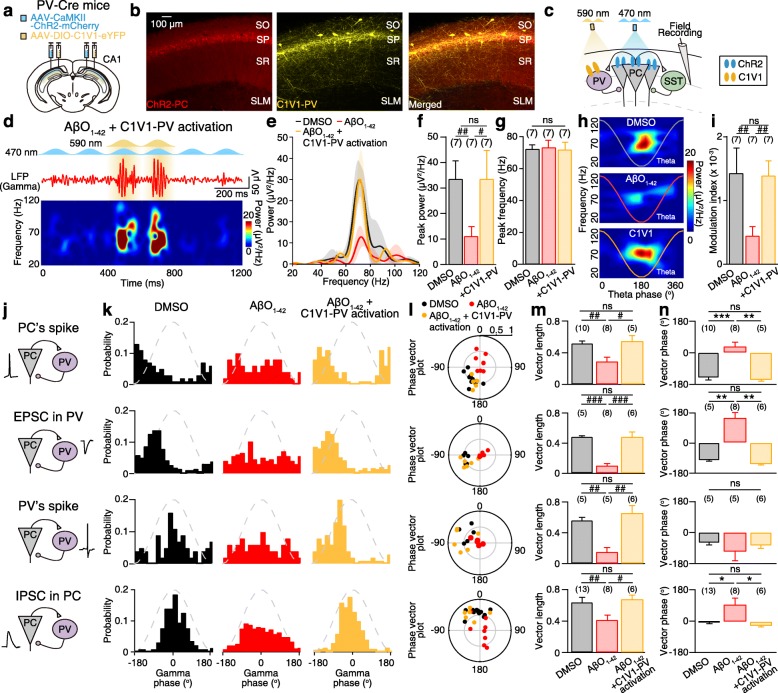
Optogenetic activation of PV interneurons restores AβO_1–42_-induced impairment of theta-nested gamma oscillations. **a** Micro-injection of AAV-CaMKII-ChR2-mCherry and AAV-DIO-C1V1-eYFP virus into CA1 area of PV-Cre mice. **b** Fluorescence image of ChR2-PC with C1V1-expressing PVs (C1V1-PV). **c** Experimental schematic. Sinusoidal (5 Hz) blue light (470 nm) and yellow light (590 nm) stimulation for activation of ChR2-PC and C1V1-PV, respectively, and field recording in CA1 area in AβO_1–42_-treated slices. **d** Sinusoidal blue and yellow light stimulation induces theta-nested gamma oscillations as shown in the band-pass filtered LFP (top) and the corresponding spectrogram (bottom), which results in the restoration of gamma oscillations in AβO_1–42_-treated slices. **e–g** Mean PSD (shade indicates SEM) of gamma oscillations (**e**), mean peak power (**f**), and mean peak frequency (**g**) of gamma oscillations in DMSO-treated slice (black), after 20-min treatment of AβO_1–42_ in the same slice (red), and with yellow light stimulation of C1V1-PV (yellow) during blue light-induced gamma oscillations. **h**, **i** Representative comodulograms showing phase-amplitude coupling of gamma oscillations to theta cycle (**h**) and mean modulation index (**i**) in each condition. **j–n** Schematic illustration of reciprocal PC-PV circuit (**j**), corresponding phase histogram (**k**), vector phases and lengths in polar plots (**l**), mean vector length (**m**), and circular mean vector phase (**n**) of CA1 PC’s spike, EPSC in PV, PV’s spike, and IPSC in CA1 PC recorded during gamma oscillations in each condition. One-way repeated-measures (**f**, **g**, **i**), one-way ANOVA with post hoc Tukey’s test (**m**, ^###^*p* < 0.001, ^##^*p* < 0.01, ^#^*p* < 0.05, ns: not significant), and Watson-Williams test (**n**, ***p < 0.001, **p < 0.01, *p < 0.05, ns: not significant). Data are represented as mean ± SEM. Data in **k**–**n** was collected from the different number of slices (DMSO 23, AβO_1–42_ 18, AβO_1–42_ + C1V1-PV 14) and animals (DMSO 17, AβO_1–42_ 10, AβO_1–42_ + C1V1-PV 8)

We then asked whether optogenetic activation of PV interneurons could rescue theta-nested gamma oscillations in AβO_1–42_-treated slices. If so, it would be strong evidence that the dysfunction of PV interneurons was the ultimate cause of reduced theta-nested gamma oscillations in AβO_1–42_-treated slices. We co-injected AAV viruses carrying ChR2 and C1V1 (AAV-DIO-C1V1-eYFP) (Fig. 4a), an opsin that opens a cation channel with peak excitation centered around green light (565 nm), in order to express ChR2 in CA1 PC and C1V1 in PV interneurons (Fig. 4b). Since green light activates ChR2-expressing PCs (Additional file 7: Figure S7), we optically stimulated C1V1-expressing PV interneurons using yellow light (590 nm), which activated C1V1-expressing PV interneurons reliably (Additional file 8: Figure S8). Using this preparation, we optically stimulated C1V1-expressing PV interneurons with yellow light in AβO_1–42_-treated slices during blue light-induced theta-nested gamma oscillations (Fig. 4c, d). PV interneuron activation successfully restored the peak power of gamma oscillations in AβO_1–42_-treated slices (Fig. 4d–f) to the level observed in DMSO-treated slices while maintaining frequency at gamma (Fig. 4g). Phase-amplitude coupling of gamma oscillations to theta cycle in AβO_1–42_-treated slices was also increased by PV interneuron activation to the level observed in DMSO-treated slices (Fig. 4h, i). Since CA1 PC spike phases relative to gamma oscillations are important for hippocampal spatial information processing [36, 37], we investigated the phase of spikes and postsynaptic currents (PSCs) relative to the gamma cycle. Following the PING model [14, 21, 23], gamma oscillations triggered the activation of CA1 PC spikes, EPSCs in PV interneurons, PV interneuron spikes, then IPSCs in CA1 PCs in sequence (Fig. 4j), with distinct phases relative to ongoing gamma cycles in DMSO-treated slices (Fig. 4k, black bars). The phase-locking of spike/synaptic current was abolished in AβO_1–42_-treated slices, making it difficult to detect a clear peak in the event phase probability (Fig. 4k, red bars). Nonetheless, optical stimulation of C1V1-expressing PV interneurons in AβO_1–42_-treated slices restored phase-locking of spikes/synaptic currents (Fig. 4k, yellow bars). The strength of phase-locking, as measured by the length of the resultant vector in the phase vector plot, was indeed restored by optical stimulation of C1V1-expressing PV interneurons (Fig. 4l, m). The mean vector phases were also rescued by optical stimulation of C1V1-expressing PV interneurons (Fig. 4n). These data show that optogenetic activation of PV interneurons restores gamma power and resynchronizes spikes/synaptic inputs to gamma cycles. This supports the idea that AβO_1–42_-induced reductions in theta-nested gamma oscillations power are caused by PV interneuron dysfunction.

### Optogenetic activation of SST interneurons restores AβO_1–42_-induced impairment of theta-nested gamma oscillation-induced tLTP

**Fig. 5:**
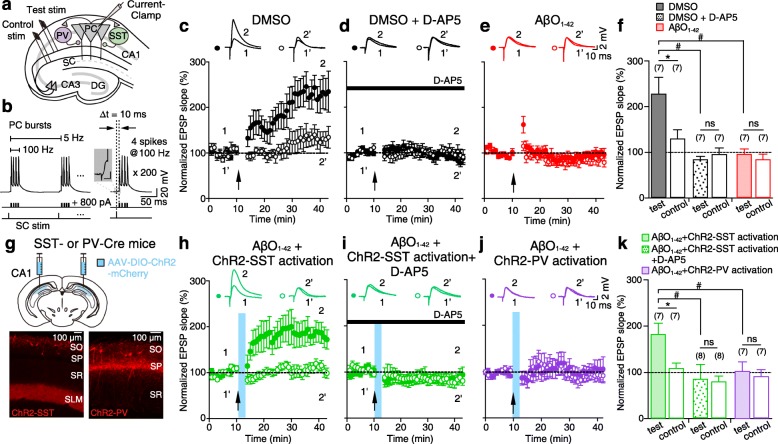
Optogenetic activation of SST interneurons restores AβO_1–42_-induced impairment of theta-nested gamma oscillation-induced tLTP. **a** Experimental schematic. Whole-cell current-clamp recordings in CA1 PC and Schaffer collateral (SC) stimulation for theta-nested gamma oscillation-like tLTP induction at CA3-CA1 excitatory synapses. **b** tLTP was induced by pairing presynaptic SC stimulation with postsynaptic CA1 PC spike bursts (4 spikes at 100 Hz) with a + 10 ms time window, repeated 200 times at 5 Hz. Inset: enlarged EPSP evoked by presynaptic SC stimulation, scale bar 10 ms, 1 mV. **c–e** EPSP slopes normalized to mean of 10-min baseline in DMSO-treated slice (**c**), + D-AP5 (50 μM) in DMSO-treated slice (**d**) and in AβO_1–42_-treated slices (**e**). Black arrow: onset of tLTP induction. Test pathways (filled circles), control pathways (empty circles). Insets: representative EPSPs at indicated time points (1, 2 or 1′, 2′). **f** Mean of normalized EPSPs slopes of last 5 min of test (filled bars) and control pathways (empty bars) in DMSO-treated slices (black), + D-AP5 in DMSO-treated slices (dotted black) and in AβO_1–42_-treated slices (red). **g** Micro-injection of AAV-DIO-ChR2-mCherry to CA1 area in SST-Cre and PV-Cre mice (top) and fluorescence images (bottom) of ChR2-expressing SST interneurons (ChR2-SST, left) and ChR2-expressing PV interneurons (ChR2-PV, right). **h–j** Same as **c–e** but tLTP induction with blue light stimulation (blue bar) for ChR2-SST activation (**h**), for ChR2-SST activation in the presence of D-AP5 (50 μM, **i**), and for activation of ChR2-PV (**j**) in AβO_1–42_-treated slices. **k** Same as **f** but with ChR2-SST activation (green), ChR2-SST activation in the presence of D-AP5 (dotted green), and ChR2-PV activation (purple) in AβO_1–42_-treated slices. Paired Student’s *t* test for comparing test and control pathways (**f**, **k**, *p < 0.05, ns: not significant), one-way ANOVA with post-hoc Tukey’s test for comparing test pathways in different conditions (**f**, **k**, ^#^*p* < 0.05). Data are represented as mean ± SEM

Theta-nested gamma oscillations have been shown to support the induction of LTP at Schaffer collateral (SC) synapses [16–19], but a direct experimental demonstration of how CA1 PCs and PV/SST interneurons partake in LTP induction at CA3-to-CA1 synapses during theta-nested gamma oscillations is lacking. To remedy this, we paired presynaptic SC stimulation-evoked excitatory postsynaptic potentials (EPSPs) with postsynaptic spike bursts (4 spikes at 100 Hz repeated at 5 Hz) at a delay (Δ*t*) of + 10 ms, thereby mimicking CA3 inputs onto CA1 PCs during theta-nested gamma oscillations (Fig. 5a, b) [38]. We found that this protocol reliably induced robust tLTP at CA3-to-CA1 synapses in DMSO-treated slices (Fig. 5c, f, black filled bar), which was NMDA receptor (NMDAR)-dependent, as it was blocked by NMDAR antagonist, D-AP5 (50 μM, Fig. 5d, f, black dotted bar). However, NMDAR-dependent tLTP was completely blocked in the AβO_1–42_-treated slices (Fig. 5e, f, red filled bar). Since PV and SST interneurons’ spikes were concurrently activated during theta-nested gamma oscillations (Fig. 2c) and by alveus stimulation of CA1 PC axons (Additional file 9: Figure S9), AβO_1–42_-induced synaptic dysfunctions of either PV or SST interneurons may have contributed to the observed tLTP impairment. To test this hypothesis, we expressed ChR2 in either SST or PV interneurons in SST-Cre or PV-Cre mice (Fig. 5g) and optically stimulated ChR2-expressing SST or PV interneurons with blue light (470 nm) during theta-nested gamma oscillation-like tLTP induction in AβO_1–42_-treated slices (Fig. 5h–j). We found that optogenetic activation of SST interneurons in AβO_1–42_-treated slices could fully restore NMDAR-dependent tLTP (Fig. 5h, k, green filled bar) that was blocked by D-AP5 (Fig. 5i, k, green dotted bar). However, optogenetic activation of PV interneurons in AβO_1–42_-treated slices could not restore tLTP (Fig. 5j, k, purple filled bar).

### AβO_1–42_ causes selective dysfunction of SST interneuron-mediated disinhibition to CA1 PC

**Fig. 6:**
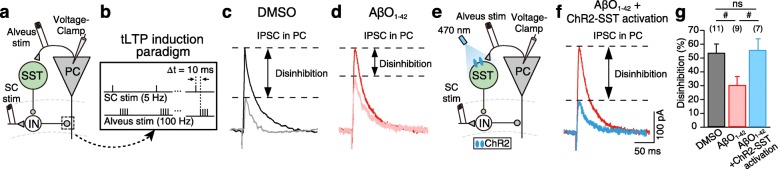
AβO_1–42_ causes dysfunction of SST interneuron-mediated disinhibition to CA1 PC. **a**, **b** Experimental setup for whole-cell voltage-clamp recordings of IPSCs in CA1 PC during theta-nested gamma oscillation-like tLTP induction. CA1 PC spikes were elicited by stimulating the CA1 PC axons in CA1 alveus. **c** IPSCs evoked by SC stimulation alone (black) and pairing of SC stimulation with alveus stimulation in DMSO-treated slices (gray). Disinhibition was measured by the difference in IPSCs amplitudes of the two conditions. **d** Same as **c** but in AβO_1–42_-treated slices. **e**, **f** Same as **a–c** but with activation of ChR2-expressing SST interneuron (ChR2-SST) with blue light (470 nm) in AβO_1–42_-treated slices. **g** Comparison of disinhibition of IPSCs amplitude in DMSO-treated (black), AβO_1–42_-treated slices (red) and with activation of ChR2-SST interneuron in AβO_1–42_-treated slices (blue). One-way ANOVA with post hoc Tukey’s test (**g**, ^#^*p* < 0.05, ns: not significant). Data are represented as mean ± SEM

How could SST activation have contributed to the restoration of NMDAR-tLTP induction during theta-nested gamma oscillations? SST interneurons, such as oriens lacunosum-moleculare (OLM) cells, inhibit the distal dendrites of PCs in CA1 [39], but they also provide disinhibition of feedforward inhibition activated by SC input to CA1 PC’s proximal dendrites [39]. Moreover, optical stimulation of SST interneuron-mediated disinhibition during LTP induction has been shown to enhance LTP [39]. Thus, one possibility is that AβO_1–42_ impairs SST interneuron-mediated disinhibition of proximal dendrites of CA1 PCs, and thereby, tLTP. To investigate this possibility, we recorded SC stimulation-evoked IPSCs from CA1 PCs and compared them with SC stimulation-evoked IPSCs paired with CA1 PC spikes evoked by alveus stimulation (4 spikes at 100 Hz, repeated at 5 Hz), which mimics theta-nested gamma oscillation-like tLTP induction, as in Fig. 5b (Fig. 6a, b, Additional file 10: Figure S10). The amplitude of SC stimulation-evoked IPSCs significantly decreased when it was paired with alveus stimulation (Fig. 6c, g, black bar), showing that SST interneurons activated by alveus stimulation resulted in SST interneuron-mediated disinhibition. SST interneuron-mediated disinhibition was significantly decreased in AβO_1–42_-treated slices (Fig. 6d, g, red bar), but it was fully restored by optical stimulation of ChR2-expressing SST interneurons to a level similar to that in DMSO-treated slices (Fig. 6e–g, blue bar). In addition, when SC stimulation was paired with 50-ms-long optical stimulation of ChR2-expressing SST interneurons alone, the amplitude of SC stimulation-evoked IPSCs was similar in both DMSO-treated and AβO_1–42_-treated slices (Additional file 11: Figure S11), further supporting our hypothesis that optical restoration of SST interneuron-mediated disinhibition underpins the restoration of tLTP induction in AβO_1–42_-treated slices.

### Distinct functional roles of PV and SST interneurons in gamma oscillogenesis and theta-nested gamma oscillation-induced tLTP

**Fig. 7:**
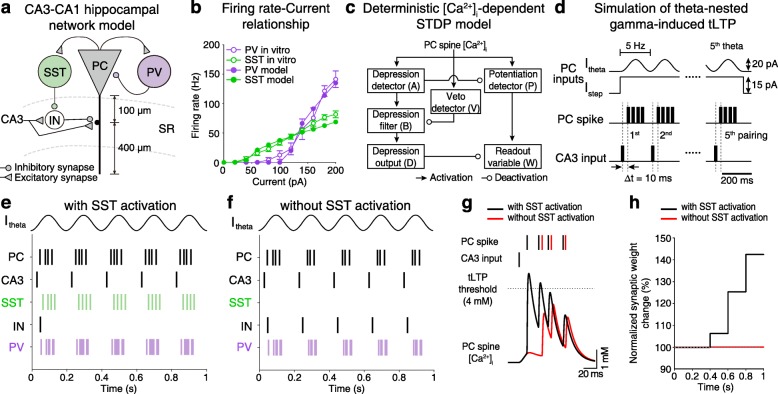
Distinct roles of PV and SST interneurons in gamma oscillogenesis and theta-nested gamma oscillation-induced tLTP. **a** Schematic diagram of CA3-CA1 hippocampal network model consisting of Hodgkin-Huxley-type computational models of CA1 PC, PV interneuron (PV model), SST interneuron (SST model), and a feedforward inhibition-mediating interneuron (IN model). The CA3 input activates IN and also provides excitation to the dendritic spine of the CA1 PC. **b** Firing rate plotted as a function of depolarizing current steps in 20 pA in PV interneuron (purple) and SST interneuron (green) recorded in vitro (empty circle, data from Additional file 4: Figure S4c, l), and that of the PV and SST models (filled circle). **c** Schematic of a deterministic [Ca^2+^]_i_-dependent spike timing-dependent plasticity (STDP) model. **d** A simulation of theta-nested gamma oscillation-induced tLTP. Oscillatory current (*I*_theta_, 5 Hz, 20 pA) superimposed with a step current (*I*_step_, 15 pA) was simulated to CA1 PC (top) to mimic gamma-frequency spikes in CA1 PC (middle). For tLTP induction, stimulation of CA3 input preceded the CA1 PC spikes by 10 ms, repeated at 5 Hz (bottom). **e**, **f** Representative raster plot of each neuron model with SST activation (**e**) or without SST activation (**f**). **g** Representative [Ca^2+^]_i_ at CA1 PC spine during tLTP induction with SST activation (black) or without SST activation (red). **h** Change in the normalized synaptic weight of CA3-CA1 synapse plotted as a function of time with (black) and without SST activation (red)

Our data supports the following hypothesis about how CA3 inputs impinging on CA1 PCs during hippocampal oscillations undergo LTP in a healthy brain [16–19]: gamma-frequency spikes of CA1 PCs during theta-nested gamma oscillations generated by perisomatic-targeting PV interneurons recruits SST interneurons, which in turn disinhibits CA1 PCs’ perisomatic dendrites, creating a window of opportunity for tLTP induction. To test this hypothesis, we built a computational network model consisting of CA1 PC, PV, and SST interneurons, together with CA3 input synapsing onto proximal dendritic spines of the CA1 PC providing feedforward inhibition to CA1 PC by activating an inhibitory interneuron (IN) (Fig. 7a). A PV interneuron was reciprocally connected to the CA1 PC while a SST interneuron disinhibited the IN. Parameters were tuned to reflect the in vitro-recorded firing rate-input current relationship (Fig. 7b, Additional file 4: Figure S4c, l). The excitatory CA3-CA1 synapse was modeled to undergo a deterministic intracellular Ca^2+^ concentration ([Ca^2+^]_i_)-dependent tLTP induction (Fig. 7c). In this model, sinusoidal 5-Hz current input that mimics blue light stimulation delivered to ChR2-expressing CA1 PC (Fig. 7d) activated the reciprocally connected PV interneuron to entrain CA1 PC and SST interneuron spikes at gamma oscillations, as shown in the spike raster plot (Fig. 7e). Such gamma-frequency-entrained SST interneuron’s spikes inhibited the IN from spiking (Fig. 7e, IN), and when CA3 input was activated at the rising phase of theta oscillations, SST interneuron-mediated disinhibition allowed the [Ca^2+^]_i_ of CA1 PC spike to cross the threshold for tLTP induction (Fig. 7g, h). In contrast, in a network model without SST interneuron (Fig. 7f), CA3 input-activated feedforward inhibition (Fig. 7f, IN) blocked tLTP induction (Fig. 7g, h). Modulation of SST interneuron activation had no effect on the entrainment of PV interneurons at gamma-frequency and phase-locking of their spikes relative to CA1 PC-generated gamma-frequency spikes (Additional file 12: Figure S12). These results further underscore the differential roles of PV and SST interneurons in hippocampal theta-nested gamma oscillations and tLTP induction, respectively, and suggest how the optogenetic activation of PV and SST could have restored gamma oscillations and tLTP in AβO_1–42_-treated slices.

## Discussion

Here we have provided the first experimental evidence on how AβO_1–42_ causes synapse-specific dysfunction in hippocampal inhibitory circuits to impair theta-nested gamma oscillations and theta-nested gamma oscillation-induced tLTP. AβO_1–42_ selectively disrupted reciprocal PC-to-PV and PV-to-PC synapses, which decreased the peak power of theta-nested gamma oscillations and desynchronized the phase of spikes and synaptic currents relative to gamma cycles (Fig. 1, 2, 3, 4). In contrast, AβO_1–42_ had no effect on either PC-to-SST synapse or SST-to-PC synapses, but it did selectively disrupt SST interneuron-mediated disinhibition to block NMDAR-mediated tLTP at CA3-to-CA1 synapses induced by theta-nested gamma oscillation-like stimulation (Figs. 5 and 6). Importantly, optical stimulation of PV and SST interneurons selectively restored theta-nested gamma oscillations and oscillation-induced tLTP, respectively, which strongly supports the conclusion that these phenomena were the result of synapse-specific dysfunctions of PV and SST interneurons induced by AβO_1–42_.

Based on our in vitro experimental observations, we built a computational network model of CA1 PC, PV, and SST interneurons which allowed us to infer possible reasons for why hippocampal oscillations are conducive to LTP in a healthy brain [16–19]. From our simulation results, we were able to see how perisomatic-targeting PV interneurons entrain both CA1 PC and SST interneurons at gamma-frequency which allowed for the SST interneuron to disinhibit CA3 input-activated feedforward inhibition onto CA1 PCs’ proximal dendrites, creating a time window for tLTP induction (Fig. 7). Thus, PV and SST interneurons have distinct functional roles in the induction of synaptic plasticity in different compartments of the CA1 PC, and the accumulation of AβO_1–42_ seen in Alzheimer’s disease may cause memory deficits due to impairment of these synaptic plasticity mechanisms.

Although all of our experiments are conducted in vitro, the gamma oscillation impairment observed in our study shares many similarities with the effects of Aβ on kainate-induced gamma oscillations in vitro [9] as well as gamma oscillations recorded in vivo in mouse models of Alzheimer’s disease [5–8]. Also, our finding that optical stimulation of PV interneurons can restore gamma oscillations is consistent with previous results showing that manipulations of PV interneurons [5, 8] or PV-like fast-spiking interneurons were able to restore gamma oscillations in Alzheimer’s disease mouse models in vivo [7]. However, unlike previous studies using animal models with the late phase of Alzheimer’s disease [5, 7, 8], the acute effects of AβO_1–42_ that we uncovered here may only account for the early phase of Alzheimer’s disease. In Alzheimer’s disease mouse models such as APP/PS1 mice [40] and hAPPJ20 mice [5], spike firing rates and membrane potentials of PV interneuron are increased while in early phase of Alzheimer’s disease, pathological effects of AβO_1–42_ are mainly limited to synaptic dysfunctions with the intrinsic neuronal properties are spared [41], which is consistent with our results (Figs. 2 and 3 and Additional file 4: Figure S4). Thus, optogenetic activation of PV interneurons could have restored theta-nested gamma oscillations by directly depolarizing PV interneurons, which in turn compensate for the AβO_1–42_-induced reduced PV interneuron-evoked EPSCs to CA1 PC (Fig. 2) to resynchronize CA1 PC spikes during theta-nested gamma oscillations (Fig. 4), consequently leading to the restoration of theta-nested gamma oscillations. In addition to the reduction in gamma oscillation power, epileptic hyper-synchronous activities are widely observed in human patients with Alzheimer’s disease [6, 42] and in genetically modified Alzheimer’s disease mouse models [5, 6, 27, 43, 44]. Since the occurrence of epileptic activities in Alzheimer’s disease mouse models requires the abnormal aggregation of Aβ fibrils [43] and tau protein [44], but not AβO_1–42_ [43], it may be that hyper-synchrony may develop with Alzheimer’s disease progression [6, 45]. In fact, it is well established that AβO_1–42_ causes hyperexcitability in excitatory neurons [26]. Also, the increase in EPSC and decrease in IPSC amplitudes in CA1 PC during kainate-induced gamma oscillations under AβO_1–42_ pathology was observed in vitro [9]. Thus, it may be that the balance between excitation and inhibition is disrupted in Alzheimer’s disease but how the same neural circuit alternates between hypo- and hyper-synchrony requires further investigation.

Although many studies manipulated PV interneurons in Alzheimer’s disease studies [5, 7, 8], our study is the first to directly show how manipulation of SST interneurons could alleviate Alzheimer’s disease-related dysfunctions. In contrast to many studies targeting dysfunctional excitatory synapses [46–49] or LTP induction-related intracellular cascades in order to restore LTP in Alzheimer’s disease mouse models [49–51], we show that reinstating SST interneuron-mediated disinhibition [39] is sufficient for restoring tLTP in AβO_1–42_-treated slices in vitro (Figs. 5 and 6). In fact, SST interneuron-mediated disinhibition unmasks the back-propagating spike required for the induction of tLTP [52, 53]. Thus, our results suggest that SST interneurons’ neural circuit dysfunction could explain the tLTP impairment caused by acute application of AβO_1–42_ resembling early stages of Alzheimer’s disease, further supported by our in silico hippocampal network simulation (Fig. 7, Additional file 12: Figure S12). Although we did not get to identify the interneuron subtype that provides disinhibition to CA1 PC through SST interneuron activation, CCK-positive interneurons such as Schaffer collateral-associated cells [54–56] or bistratified cells [39] that are located in the stratum radiatum could be potential candidates. Thus, identifying the interneuron subtypes involved in disinhibition could help target the disinhibitory synapse that is impaired by AβO_1–42_ pathology. A recent study reported that optogenetic activation of OLM interneurons can induce type 2 theta oscillations in vivo [31], indicating that SST interneurons may also contribute to the generation of theta oscillations in addition to providing disinhibition to CA1 PC in vivo. Since we optically stimulated theta oscillations in order to induce gamma oscillations in vitro, our data cannot resolve the individual contribution of PV or SST interneurons on theta oscillation impairment in Alzheimer’s disease [57, 58]. Moreover, it is possible that theta-nested gamma oscillations could play a role in the induction of synaptic plasticity in interneurons [59]; thus, the neural circuit mechanism linking theta-nested gamma oscillations and tLTP may be more intricate than suggested in the present study (Fig. 7). Interestingly, a recent study reported re-emergence of LTP in aged Tg2576 Alzheimer’s disease mice which correlates with a decrease in PV interneuron number [60]. Thus, the specific manner in which PV and SST interneurons are affected as the pathologies of Alzheimer’s disease progress with age in vivo to disrupt synaptic plasticity requires further investigation. Nonetheless, our data suggests that targeted manipulation of interneuron populations in the hippocampus may be a promising approach for treatments of early-stage Alzheimer’s disease.

Although the optogenetic manipulation technique we adopted in this study targeted CA1 PV and SST interneurons, in CA1 alone, there are more than 20 interneuron subtypes [61, 62] and PV and SST interneurons do not relate to specific interneuron types, nor indeed are these two markers entirely non-overlapping in CA1 [63–68]. PV can be expressed in both axo-axonic and fast-spiking interneurons, and SST can be found not only in oriens lacunosum-moleculare interneurons, but in various long-range projecting interneurons, too. Indeed, bistratified cells (found in stratum oriens) express both PV and SST [54, 69–71]. Therefore, care is warranted in interpreting our results.

## Conclusions

In summary, by optogenetically manipulating PV and SST interneurons, here we showed for the first time that AβO_1–42_ causes synapse-specific dysfunctions in PV and SST interneurons’ synapses, which allows us to uncover how AβO_1–42_ causes concomitant impairments of hippocampal theta-nested gamma oscillations and oscillation-induced tLTP at CA3-to-CA1 synapses. Thus, our findings provide crucial insight that will help guide future studies aimed at identifying the molecular target that gives rise to AβO_1–42_-induced synapse-specific dysfunctions, potentially leading to novel therapeutic targets for Alzheimer’s disease.

## Methods

### Animals

Three different lines of mice, C57BL/6 mice, PV-Cre knock-in mice (C57BL/6 background, Jackson Laboratory, stock #017320), and SST-IRES-Cre (C57BL/6 background, Jackson Laboratory, stock #013044) knock-in mice (4–11 weeks old) were used [72]. All animals were kept in 12:12-h light-dark cycles with food and water available ad libitum. All animal care and experimental procedures were approved by the Institutional Animal Care and Use Committee of Korea University (KUIACUC-2017-112).

### Virus

AAV particles were purchased from the UNC Vector Core. To express ChR2 [73] selectively in CA1 PC, AAV5-CaMKII-hChR2(E123T/T159C)-p2A-mCherry-WPRE (3.8 × 10^12^ virus molecules/ml, 1 μl) was injected in all three different lines of mice bilaterally into the hippocampus. For the selective expression of eYFP, Arch, ChR2, or C1V1 on PV or SST interneurons, AAV2-EF1a-DIO-EYFP (4.6 × 10^12^ virus molecules/ml, 1 μl), AAV5-EF1a-DIO-eArch3.0-EYFP (5 × 10^12^ virus molecules/ml, 1 μl), AAV5-EF1a-DIO-hChR2(E123T/T159C)-p2A-mCherry-WPRE (3.8 × 10^12^ virus molecules/ml, 1 μl), or AAV2-EF1a-DIO-C1V1(E162T)-TS-p2A-EYFP-WPRE (3 × 10^12^ virus molecules/ml, 1 μl) were injected bilaterally into the hippocampus of in PV-Cre or SST-Cre mice.

### Stereotaxic virus injections

Mice were deeply anesthetized under 2% isoflurane (2 ml/min flow rate) and head-fixed into a stereotaxic frame (Stoelting Co.). Craniotomies were made bilaterally to target CA1 area of the hippocampus for viral injections (from bregma: anteroposterior − 2.70 mm, lateral ± 2.50 mm, and dorsoventral − 1.75 mm or anteroposterior − 2.56 mm, lateral ± 2.6 mm, and dorsoventral − 1.85 mm). One microliter of each virus suspension was injected into the CA1 area of the hippocampus at a rate of 0.15 μl/min through a Hamilton syringe using a motorized stereotaxic injector (Stoetling Co.). The syringe was left in the brain for more than 5 min to allow for virus diffusion. The scalp was sutured and disinfected with antibiotic, after which the mice were returned to their home cage for recovery for at least 14 days.

### Preparation and treatment of AβO_1–42_ to hippocampal slices

Soluble AβO_1–42_ was prepared following methods in Lambert et al. [4] with a slight modification [74]. Aβ_1–42_ or Aβ_42–1_ powder (Bachem) was dissolved in 1,1,1,3,3,3-hexafluoro-2-propanol (HFIP, Sigma Aldrich) for monomerization at a final concentration of 1 mM and incubated for 90 min. HFIP was evaporated under vacuum condition (SpeedVac). The remaining thin and clear film of Aβ_1–42_ or Aβ_42–1_ was dissolved in dimethyl sulfoxide (DMSO, Sigma Aldrich) to make 5 mM Aβ_1–42_ or Aβ_42–1_ stock, which was aliquoted and frozen at − 20 °C. The Aβ_1–42_ or Aβ_42–1_ stock was thawed and diluted to 100 μM in artificial cerebrospinal fluid (aCSF, containing (in mM): 126 NaCl, 3 KCl, 1.25 NaH_2_PO_4_, 2 MgSO_4_, 2 CaCl_2_, 25 NaHCO_3_, and 10 glucose at pH 7.2–7.4 bubbled with 95% O_2_/5% CO_2_). After dilution, Aβ_1–42_ or Aβ_42–1_ solution was incubated for 18 h at 4 °C for Aβ oligomerization. Before the recording, 2% DMSO (vehicle) and 100 μM AβO_1–42_ or AβO_42–1_ were treated into hippocampal slices in 31.2 ml of aCSF for 20 min by diluting it to a final concentration of 200 nM AβO_1–42_ or AβO_42–1_ in 0.004% DMSO for each condition.

### Western blot analysis

#### Sodium dodecyl sulfate-polyacrylamide gel electrophoresis (*SDS-PAGE*)

AβO_1–42_ were prepared as described above and resolved on a nonreducing 4–15% tris-glycine–SDS-PAGE gels with LDS sample buffers [75]. The gel was transferred on to a 0.2-μm PVDF membrane (Bio-Rad) according to the manufacturer’s recommendation. Membranes were blocked in 5% bovine serum albumin (BSA) in tris-buffered saline containing 0.01% Tween 20 for 1 h at room temperature. Blots were incubated in the primary antibody mOC64 (rabbit monoclonal against amino acid residues 3–6 of Aβ; Cat# ab201060, Lot# GR3235744-4, RRID: AB_2818982, Abcam) [76] at 1:200 dilution overnight at 4 °C. Immunoreactivity was detected with enhanced chemiluminescence (Bio-Rad) and imaged using Fluorchem E system (ProteinSimple). Molecular weight values were estimated using Precision Plus Protein™ Dual Color Standards (Bio-rad).

#### Native PAGE

AβO sample was diluted with native PAGE sample buffer (Bio-rad) and then subjected to native PAGE using a 4–15% tris-glycine gel with the tris-glycine running buffer (Bio-rad). Following transfer to PVDF membrane, membranes were blocked in 5% BSA in Tris-buffered saline containing 0.01% Tween 20 for 1 h at room temperature. Blots were probed using rabbit monoclonal Aβ antibody (mOC64, 1:200, Cat# ab201060, Lot# GR3235744-4, RRID: AB_2818982, Abcam) overnight at 4 °C. Immunoreactivity and imaging were performed as described above.

### In vitro hippocampal slice preparation

Mice were deeply anesthetized using 1.25% Avertin solution (8 g of 2, 2, 2-Tribromoethanol and 5.1 ml of 2-methyl-2-butanol in 402.9 ml saline, Sigma Aldrich) at a delivery rate of 0.2 ml/10 g body weight and perfused with ice-cold cutting solution (containing (in mM): 180 sucrose, 2.5 KCl, 1.25 NaH_2_PO_4_, 25 NaHCO_3_, 11 glucose, 2 MgSO_4_, and 1 CaCl_2_ at pH 7.2–7.4 oxygenated with 95% O_2_/5% CO_2_). Either coronal or horizontal hippocampal slices (300–400 μm) were cut using a vibratome (VT 1000 S, Leica Microsystems). Slices were allowed to recover for 20 min in a mixture of cutting solution and aCSF solution at 1:1 ratio, after which the slices were further incubated in aCSF for at least 1 h at 30–32 °C before performing electrophysiological recordings. To compare between DMSO and AβO_1–42_ conditions in the same slice (Fig. 1, Fig. 4c–i), hippocampal slice was first treated with 2% DMSO in aCSF for 20 min and then the same hippocampal slice was treated with 100 μM AβO_1–42_ or AβO_42–1_ in aCSF by diluting to a final concentration of 200 nM for 20 min. In all other experiments (Figs. 2, 3, 5, and 6 and Additional file 3: Figure S3, Additional file 4: Figure S4, and Additional file 11: Figure S11), hippocampal slices were treated with either 2% DMSO or 100 μM AβO_1–42_ or AβO_42–1_ in aCSF by diluting to a final concentration of 200 nM for 20 min before performing electrophysiological recordings.

### In vitro field and patch-clamp recordings

Slices were moved to a recording chamber filled with aCSF (30–32 °C), and CA1 area of the hippocampus was identified under the guidance of differential interference contrast microscopy (BW51W, Olympus). LFP was recorded in the CA1 PC layer using a borosilicate glass electrode (2–4 MΩ) filled with aCSF (Figs. 1, 2, 3, and 4 and Additional file 2: Figure S2, Additional file 3: Figure S3, Additional file 5: Figure S5, Additional file 6: Figure S6, and Additional file 7: Figure S7). In some experiments (Figs. 2c–h, 3a–g, and 4j–n), LFP recordings were simultaneously performed with whole-cell patch-clamp recordings from either CA1 PC, PV, or SST interneurons using borosilicate glass electrode (4–8 MΩ) in either voltage-clamp or current-clamp mode. All synaptic currents were recorded in voltage-clamp recordings with electrodes filled with internal solution containing (in mM) 115 Cesium methanesulfonate (CsMSF), 8 NaCl, 10 HEPES, 0.3 GTP-NaCl, 4 ATP-Mg, 0.3 EGTA, 5 QX-314, and 10 BAPTA (pH 7.3–7.4 and 280–290 mOsm/L). IPSC and EPSC were recorded at the holding potential of + 10 mV and − 80 mV, respectively. In recording spikes and intrinsic membrane properties in current-clamp recordings, electrodes were filled with intracellular solution containing (in mM) 110 K-gluconate, 40 HEPES, 4 NaCl, 4 ATP-Mg, and 0.3 GTP-NaCl (pH 7.2–7.3 and 270–300 mOsm/L). Intrinsic membrane properties such as spike probability, sag, and rebound potential were measured at resting membrane potential of the neuron in response to current steps (0 pA to ± 200 pA for 500 ms in 20 pA steps). Input resistance (MΩ) and membrane time constant (*τ*) were analyzed based on the voltage response to 50-ms-long negative current step (5 pA) by fitting an exponential curve,

$$ {R}_{\mathrm{in}}=\frac{\left({V}_0-{V}_{\mathrm{steady}}\right)}{I} $$

$$ V={V}_0+{Ae}^{\left(-\frac{t}{\tau}\right)} $$

where *V*_0_ is the initial voltage, *V*_steady_ is the steady state voltage of the first exponential curve fit, *A* is the amplitude constant, and *I* is the amplitude of the current step. To record EPSCs evoked by PCs in PV or SST interneurons, a stimulation electrode was placed in the alveus on the subiculum side of the CA1 area to stimulate the axons of PC with a radial cut made between CA1 and subiculum to block the activation of CA3 axons (Fig. 2i–p). To analyze the S-R curve of PC-evoked EPSCs in PV or SST interneurons, alveus was stimulated using a single electrical stimulation pulse (100 μs) at six different intensities (10, 50, 100, 150, 200, and 300 μA, Fig. 2j, n). The alveus stimulation intensity which gave 50% of the maximal EPSC response (half-maximal stimulus, 115–210 μA) was used in subsequent experiments measuring PPR and short-term plasticity, for which a train of ten stimulation pulses at 50 Hz (100 μs; 115–210 μA) were delivered (Fig. 2k, o). Total charge of PC-evoked EPSCs was calculated by integrating the area under the EPSC trains (Fig. 2l, p). All signals were amplified (MultiClamp 700B amplifier, Molecular Devices), low-pass filtered at 10 kHz, and acquired at 5 kHz using ITC-18 data acquisition interface (HEKA Elektronik). Igor Pro software (WaveMetrics) was used for generating command signals, acquiring data as well as data analysis. In current-clamp recordings, only cells with resting membrane potential negative to − 50 mV and with input resistance in the range of 100–400 MΩ were included in the analysis. Reported voltages are corrected for the liquid junction potential, which was calculated as ~ 10 mV. In voltage-clamp recordings, 10 min was allowed after break-through for stabilization before recordings commenced. Series and input resistance were monitored throughout the experiment, and cells with > 20% change in series resistance were discarded.

### Light-induced theta-nested gamma oscillations and gamma phase analysis

For the induction of theta-nested gamma oscillations, ChR2-expressing PCs were activated by sinusoidal (5 Hz) blue light (470 nm) [34] (Fig. 1, 2, 3, and 4 and Additional file 2: Figure S2, Additional file 3: Figure S3, Additional file 5: Figure S5, Additional file 6: Figure S6, and Additional file 7: Figure S7). Blue light was delivered using a digital micromirror device (DMD, Polygon400, Mightex) through the objective (× 40) of the microscope (BX51W, Olympus), which covered the 550-μm diameter circle of the CA1 area with the center of the illumination positioned at the field electrode. The intensity of the blue light varied between 0 to a maximum intensity of 15 mW, which was controlled using a custom-made Arduino-based controller. Igor Pro was used to control DMD and synchronize optical stimulation with the electrophysiological recordings. LFP data were first down-sampled to 1 kHz and band-pass filtered between 20 and 120 Hz for gamma oscillations. Welch’s power spectral densities (PSD) of gamma oscillations (3 repetitions of 1-s theta-nested gamma oscillations) were analyzed to quantify the peak power and peak frequency (Figs. 1h–j and 4e–g and Additional file 2: Figure S2, Additional file 3: Figure S3, Additional file 5: Figure S5, Additional file 6: Figure S6, and Additional file 7: Figure S7). Spectrogram of gamma oscillations was generated using short-time Fourier transform with window size = 100 ms and step size = 1 ms. Phase histogram (Fig. 4k) of spike or PSC was generated by calculating the instantaneous phase of spikes or PSCs using the Hilbert transform of simultaneously recorded gamma oscillations. The zero phase of gamma oscillations was defined as the peak of the gamma cycle. Probability of spike or PSCs as a function of the phase of reference gamma oscillations was obtained using 20 bins. Resultant vectors were calculated from the phase histogram and plotted in the polar plot (Fig. 4l) from which vector length (Fig. 4m) and vector phase (Fig. 4n) were calculated. Mean value and statistical significance of vector phase were calculated using the Circular Statistics Toolbox in MATLAB (R2018a) [77]. To generate phase-amplitude comodulograms of theta-nested gamma oscillations (Figs. 1k and 4h and Additional file 3: Figure S3, Additional file 5: Figure S5, and Additional file 6: Figure S6), theta phase was calculated using Hilbert transformation and binned into 20 phase bins with 18° intervals. At each theta bin, the power spectrogram of gamma oscillations was calculated using short-time Fourier transform. The zero phase of theta oscillations was defined as the peak of the theta cycle. To analyze the phase-amplitude coupling strength of theta-nested gamma oscillations (Figs. 1l, 4i, Additional file 3: Figure S3, Additional file 5: Figure S5 and Additional file 6: Figure S6), we calculated the modulation index which is defined as the normalized Kullback-Leibler distance between probability distribution of gamma amplitude per each theta phase bin (18 bins with 20° intervals) and uniform distribution [78]. To obtain the probability distribution of gamma amplitude, mean amplitude of gamma oscillations for each bin was normalized by the sum of gamma amplitude of total bins. Modulation index value of 0 indicates the absence of phase-amplitude coupling, and the higher modulation index value indicates the stronger phase-amplitude coupling.

### Optical modulation of opsin-expressing PV and SST interneurons during patch-clamp recordings

We expressed Arch or C1V1 in PV and SST interneurons and ChR2 in PC in the same hippocampal slice to optically inactivate (Fig. 3b–e, Additional file 5: Figure S5, and Additional file 6: Figure S6) or activate (Fig. 4a–d) interneurons during theta-nested gamma oscillations, respectively. The optimal wavelength for stimulating Arch is a green-colored 565-nm light. However, since 565-nm green light also induced excitatory synaptic currents by activating ChR2-expressing PCs (Additional file 7: Figure S7b, d) as well as inducing gamma oscillations in the LFP (Additional file 7: Figure S7b, e) while 590-nm yellow light had no direct effect on ChR2-expressing PC (Additional file 7: Figure S7c, d), we used 590-nm yellow light in activating both Arch- and C1V1-expressing interneurons during blue light-induced theta-nested gamma oscillations. The effectiveness of 590-nm yellow light on Arch-expressing PV and SST interneurons was tested by performing whole-cell voltage-clamp recordings in PV-Cre or SST-Cre mice, respectively (Additional file 8: Figure S8). For the inactivation of Arch-expressing interneurons during theta-nested gamma oscillations (Fig. 3d, e, Additional file 5: Figure S6, and Additional file 6: Figure S6), a tonic yellow light of a fixed light intensity (1 s, 3 mW) was delivered using the DMD. For the activation of C1V1-expressing PV interneuron during theta-nested gamma oscillations (Fig. 4c, d), a sinusoidal (5 Hz) yellow light (590 nm) was delivered through DMD with the intensity of light sinusoidally varied between 0 and 3 mW using a custom-made Arduino-based controller. To record IPSC evoked by PV and SST interneurons in CA1 PC, ChR2-expressing PV and SST interneurons were optically stimulated with blue light (470 nm) in PV-Cre and SST-Cre mice, respectively, during whole-cell voltage-clamp recordings with the membrane held at + 10 mV (Fig. 3i, n). To analyze the S-R curve of PV/SST interneuron-evoked IPSCs in CA1 PC, a single light pulse (470 nm, 5 ms) was delivered to ChR2-expressing PV or SST interneurons at different light powers (5, 10, 25, 50, 75, 100% of maximal light power (15 mW), Fig. 3j, o). The light power which gave 50% of the maximal IPSC response (half-maximal stimulus, 3.75–9 mW) was used for the subsequent PPR and short-term plasticity analysis, for which a train of ten blue light pulses at 50 Hz were delivered (470-nm light, 5-ms duration, Fig. 3k, p; 3.75–9 mW). The total charge of PV/SST-evoked IPSCs was calculated by integrating the area under the IPSC train (Fig. 3l, q).

### Theta-nested gamma oscillation-induced tLTP induction protocol

In order to induce theta-nested gamma oscillation-induced tLTP at CA3-CA1 synapse during theta-nested gamma oscillation-like activity, we paired the presynaptic EPSP evoked by SC stimulation with postsynaptic bursts (4 spikes at 100 Hz, each spike elicited with 3 ms current steps, 800 pA) with a 10-ms time window repeated at 5 Hz [38] for 200 times. EPSPs were evoked every 6 s using two stimulating electrodes placed in the stratum radiatum of the CA1 area to activate SC, one for monitoring EPSPs in the control pathway and one for test pathway (Fig. 5a, b). Test and control pathways were stimulated 2 s apart. EPSP amplitudes were in the range of 3–5 mV (150–400 μA, 20–80 μs, Digitimer Ltd.) and were recorded at membrane voltage held at − 75 mV. Following 10 min of baseline EPSP recordings of both pathways, tLTP induction protocol was delivered to the test pathway, after which EPSPs were evoked every 6 s in both pathways in either DMSO-treated or AβO_1–42_-treated hippocampal slices prepared from C57BL/6 mice (Fig. 5c–e). To investigate the effect of activation of PV and SST interneurons on tLTP in AβO_1–42_-treated hippocampal slices, we expressed ChR2 in either PV or SST interneurons and optically stimulated ChR2-expressing PV or SST interneurons using tonic blue light (470 nm, X-cite 110LED, Excelitas Tech., 100% light intensity) during the tLTP induction in AβO_1–42_-treated hippocampal slices prepared from PV-Cre or SST-Cre mice, respectively (Fig. 5g–j). tLTP induction was repeated in the presence of 50 μM D-AP5 to see if the tLTP is NMDA receptor-dependent (Fig. 5d, i). The slope of EPSP was calculated as an index of synaptic efficacy, measured by performing a linear fit on the rising slope of the EPSP between time points corresponding to 20 and 80% of the EPSP peak amplitude. Changes in synaptic efficacy were estimated as percentage change relative to the mean EPSP slope during the first 10 min of baseline recordings. To compare synaptic efficacy between neurons and experimental conditions, the mean of the normalized EPSP slope in the time period between 25 and 30 min after the tLTP induction was calculated (Fig. 5f, k).

### SST interneuron-mediated disinhibition

To measure SST interneuron-mediated disinhibition during tLTP induction, we performed whole-cell voltage-clamp recordings in PC to record SC stimulation-evoked IPSC before and during tLTP induction. tLTP induction was performed by pairing of presynaptic EPSP and postsynaptic PC spikes by stimulating the SC and evoking postsynaptic spikes by stimulating the CA1 axons in the alveus at 100 Hz (4 pulses) with 10-ms time window, repeated at 5 Hz for 20 times (Fig. 6b, Additional file 10: Figure S10). All recordings were performed in the presence of D-AP5 (50 μM) to prevent synaptic plasticity during tLTP induction. To test if alveus stimulation can elicit spikes in PV and SST interneurons similar to that during blue light-induced theta-nested gamma oscillations as in Fig. 2c, we performed current-clamp recordings in PV and SST interneurons and stimulated alveus at 100 Hz (4 stimuli) repeated at 5 Hz (Additional file 9: Figure S9b, d, top). To ensure that alveus stimulation activated PC axons and is not a result of direct stimulation of other pathways, we repeated the experiments in the presence of D-AP5 (50 μM) and CNQX (20 μM) to block NMDA and AMPA receptors (Additional file 9: Figure S9b, d, bottom). Since alveus stimulation can activate both PV and SST interneurons to provide direct inhibition to PC, we isolated the SC stimulated IPSC during tLTP induction (Additional file 10: Figure S10b, (4), gray) by subtracting the IPSC evoked by alveus stimulation alone (Additional file 10: Figure S10b, (2) Alveus stim, light brown) from the IPSC evoked by pairing SC stimulation with alveus stimulation (Additional file 10: Figure S10b, (3) SC + alveus stim, brown). In calculating the SST interneuron-mediated disinhibition, we took the difference between the IPSC amplitude evoked by SC stimulation alone (Additional file 10: Figure S10b, (1) SC stim, black) and IPSC amplitude calculated in (4) (Additional file 10: Figure S10b, gray). In order to directly test the effect of the activation of SST interneurons on SC stimulation-evoked IPSC, we optically activated ChR2-expressing SST interneurons simultaneously with SC stimulation in the DMSO-treated and AβO_1–42_-treated hippocampal slices prepared from SST-Cre mice (Additional file 11: Figure S11).

### Drugs

CNQX, SR95531 (GABAzine), and D-AP5 were purchased from Tocris. PBS, Urea, and Aβ_1–42_/Aβ_42–1_ powder were purchased from Gibco, Affymetix, and Bachem, respectively. DMSO and the other regents were all purchased from Sigma. For western blot analysis, rabbit monoclonal antibody mOC64was purchased from Abcam (Cat# ab201060, Lot# GR3235744-4, RRID: AB_2818982). Horseradish peroxidase (HRP)-conjugated anti-rabbit antibodies (Cat# 170-6515, Control# 64170140, RRID: AB_2617112), Mini-PROTEAN TGX 4–15% tris-glycine gels, 4x Laemmli sample buffer, Native sample buffer, and running buffer were all purchased from Bio-Rad.

### Fluorescence imaging

To confirm the expression of opsins in PC, PV, and SST interneurons, hippocampal slices were post-fixed overnight in 4% paraformaldehyde at 4 °C and subsequently washed in PBS. Washed slices were mounted with CUBIC mount solution [79], a tissue clearing technique that removes lipids from the sample to enhance transparency in imaging. Images were acquired using a confocal microscope (LSM-700, ZEISS) under a × 10 and × 20 objective.

### CA3-CA1 hippocampal network model

To test whether SST interneuron-mediated disinhibition is required for the theta-nested gamma oscillation-induced tLTP at CA3-CA1 synapse in a computational model, we modeled CA3-CA1 hippocampal network consisted of a multi-compartment PC, single-compartment PV interneuron (PV model), SST interneuron (SST model), and a feedforward inhibition-mediating interneuron (IN model) as the Hodgkin-Huxley neuron model [80] (Fig. 7a). The PC model was composed of a soma, an apical dendrite, and a dendritic spine, containing leakage (g_L_), Na^+^ (g_Na_), delayed-rectifier K^+^ (g_KDR_), A-type K^+^ (g_A_), L-type Ca^2+^ (g_CaL_), M-type K^+^ (g_KM_), afterhyperpolarization-activated (g_AHP_), and hyperpolarization-activated (g_h_) channels. PV, SST, and IN models contain leakage (g_L_), Na^+^ (g_Na_), delayed-rectifier K^+^ (g_KDR_), and A-type K^+^ (g_A_) channels. Spike activities of PV and SST models were calibrated to replicate the in vitro-measured firing rate-current relationship (Fig. 7b, Additional file 4: Figure S4c, l). All morphological, passive, and active parameters of models are shown in Additional file 13: Table S1. CA3-CA1 synapse was modeled at the PC spine located at 100 μm from PC soma. CA3 input evoked an EPSP in PC through AMPA and NMDA receptor models. AMPA receptor was modeled as a single-exponential model, and NMDA receptor was modeled with voltage-dependent magnesium block using the following equations,

$$ {I}_{\mathrm{AMPA}}={g}_{\mathrm{AMPA}}\times \left({e}^{-\frac{t}{\tau }}\right)\times \left({V}_m-{E}_{\mathrm{AMPA}}\right),\kern0.5em {I}_{\mathrm{NMDA}}={g}_{\mathrm{NMDA}}\times \left({e}^{-\frac{t}{\tau_{\mathrm{rise}}}}-{e}^{-\frac{t}{\tau_{\mathrm{decay}}}}\right)\times \left({V}_m-{E}_{\mathrm{NMDA}}\right)/\Big(1+\left(\frac{\left[ mg\right]}{n}\right)\times {e}^{-\tau}\times {V}_m $$

where *V*_*m*_ is the membrane potential, *I* is the synaptic current, *g* is the maximal conductance (AMPA, 0.3 pS; NMDA, 1 nS), *τ* is time constants (AMPA, 7 ms; *τ*_rise_ for NMDA, 4 ms; *τ*_decay_ for NMDA. 21 ms), *E* is the reversal potential (0 mV), and [mg] is the magnesium concentration (0.5 mM). Maximal conductance of AMPA and NMDA was modeled to fit AMPA/NMDA ratio recorded in vitro [81]. Excitatory and inhibitory synapses between PC, PV, SST, and IN models were modeled using a double-exponential model [82]. All excitatory and inhibitory synapses had *τ*_rise_ of 3 ms and *τ*_decay_ of 15 ms and 40 ms, respectively. For tLTP simulation, we used a deterministic Ca^2+^-dependent STDP model (Fig. 7c) [83]. tLTP was considered to be induced when intracellular Ca^2+^ concentration ([Ca^2+^]_i_) is greater than 4 μM which triggered a potentiation detector (*P*). Synaptic weight of CA3-CA1 AMPA synapse was determined by the readout variable (*W*). To simulate theta-nested gamma oscillation-induced spikes in PC, we injected oscillatory current (5 Hz, 20 pA) superimposed with a tonic step current (15 pA) onto PC soma. For tLTP induction, we paired CA3 input with PC spikes with a time window of 10 ms (*Δt*, Fig. 7d). The pairing was repeated five times, and all parameters of the STDP model are listed in Additional file 14: Table S2. In order to investigate whether the presence of SST interneurons in the network model has any effect on the entrainment of PV interneuronal spikes at gamma-frequency, firing rates of PC and PV were calculated for the first and the successive theta cycles (Additional file 12: Figure S12a, b). Also, the spike phases of PV interneurons were calculated relative to the PC spike timing where the inter-spike interval of PC spikes were considered as a period of gamma-frequency and each spike was considered as the trough of gamma cycle (Additional file 12: Figure S12c, d). All simulations were repeated 10 times with Gaussian white noise that generated membrane voltage fluctuations (*σ* = 50 pA, peak-to-peak amplitude of fluctuation = ~ 5 mV, [84]). All simulations were performed using the NEURON simulator [85] with a sampling rate of 10 kHz. The model is available on GitHub (https://github.com/kuncl/thetagamma_tLTP).

### Data analysis

All data analysis was conducted using Igor Pro or MATLAB with custom-written scripts. Excel (Microsoft) and SPSS (IBM) software were used for statistical analyses.

### Statistical analysis

Data are represented as mean with individual data values or mean ± SEM. Statistical significance was measured using Student’s *t* test or one-way, one-way repeated-measures, and two-way ANOVA followed by post hoc Tukey’s test. *p* value less than 0.05 was considered statistically significant. Statistical significance of spike phases was tested using Watson-Williams multi-sample circular test [86].

## Supplementary information

**Additional file 1 : Figure S1.** Western blot of native PAGE showing AβO_1–42_ after incubation at 4 °C for 18 h.

**Additional file 2 : Figure S2.** Stability of optogenetically-induced theta-nested gamma oscillations in hippocampal slices in vitro.

**Additional file 3 : Figure S3.** Impairment of optogenetically-induced theta-nested gamma oscillations in AβO_1–42_-treated hippocampal slice in vitro.

**Additional file 4 : Figure S4.** Intrinsic properties of SST and PV interneurons in DMSO- and AβO_1–42_-treated hippocampal slices in vitro.

**Additional file 5 : Figure S5.** Optogenetic inactivation of Arch-expressing PV interneurons reduces the power of gamma oscillations.

**Additional file 6 : Figure S6.** Optogenetic inactivation of Arch-expressing SST interneurons has no effect on the power of gamma oscillations.

**Additional file 7 : Figure S7.** Response of ChR2-expressing PC to different wavelengths of sinusoidal light stimuli.

**Additional file 8 : Figure S8.** Current response of Arch-expressing PV interneuron, Arch-expressing SST interneuron, and C1V1-expressing PV interneuron to 590 nm light stimulation.

**Additional file 9 : Figure S9.** Stimulation of CA1 PC axons with a theta-nested gamma oscillation-like pattern entrains PV and SST interneurons at gamma frequency.

**Additional file 10 : Figure S10.** Experimental protocol for measuring SST interneuron-mediated disinhibition.

**Additional file 11 : Figure S11.** Optical stimulation of ChR2-expressing SST interneurons restores AβO_1–42_-induced impairment of SST interneuron-mediated disinhibition.

**Additional file 12 : Figure S12.** The effect of SST interneuron activation on spike firing rates and spike phases of CA1 PC and PV interneurons during theta-nested gamma oscillations in silico.

**Additional file 13 : Table S1.** Parameters of CA1 PC, PV, SST and IN models.

**Additional file 14 : Table S2.** Parameters of the deterministic Ca^2+^-dependent STDP model.

## Data Availability

All data generated during this study are included in either the manuscript or its additional files.
