# Supplementary figures and images for: Optogenetic activation of parvalbumin and somatostatin interneurons selectively restores theta-nested gamma oscillations and oscillation-induced spike timing-dependent long-term potentiation impaired by amyloid β oligomers
Source: BMC Biol. 2020 Jan 15;18:7. doi: 10.1186/s12915-019-0732-7 (PMC6961381; doi:10.1186/s12915-019-0732-7)

**Additional file 1**


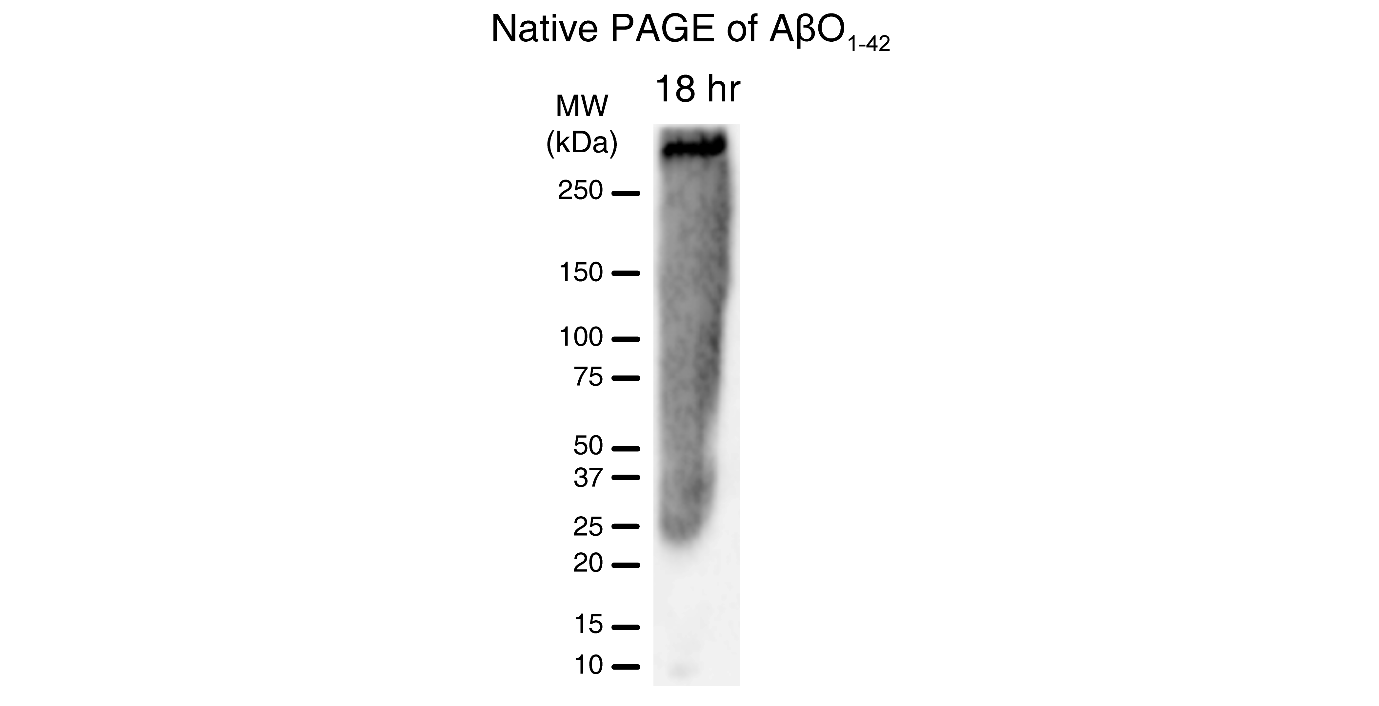


**Figure S1.** Western blot of native PAGE showing AβO1-42 after incubation at 4 °C for 18 hr.

Supplement: Supplementary file 1 — Additional file 1 : Figure S1. Western blot of native PAGE showing AβO1–42 after incubation at 4 °C for 18 h. [file 12915_2019_732_MOESM1_ESM.docx]
