# Supplementary material for: Optogenetic activation of parvalbumin and somatostatin interneurons selectively restores theta-nested gamma oscillations and oscillation-induced spike timing-dependent long-term potentiation impaired by amyloid β oligomers
Source: BMC Biol. 2020 Jan 15;18:7. doi: 10.1186/s12915-019-0732-7 (PMC6961381; doi:10.1186/s12915-019-0732-7)
Supplement: Supplementary file 2 — Additional file 2 : Figure S2. Stability of optogenetically-induced theta-nested gamma oscillations in hippocampal slices in vitro. [file 12915_2019_732_MOESM2_ESM.docx]

**Additional file 2**

**
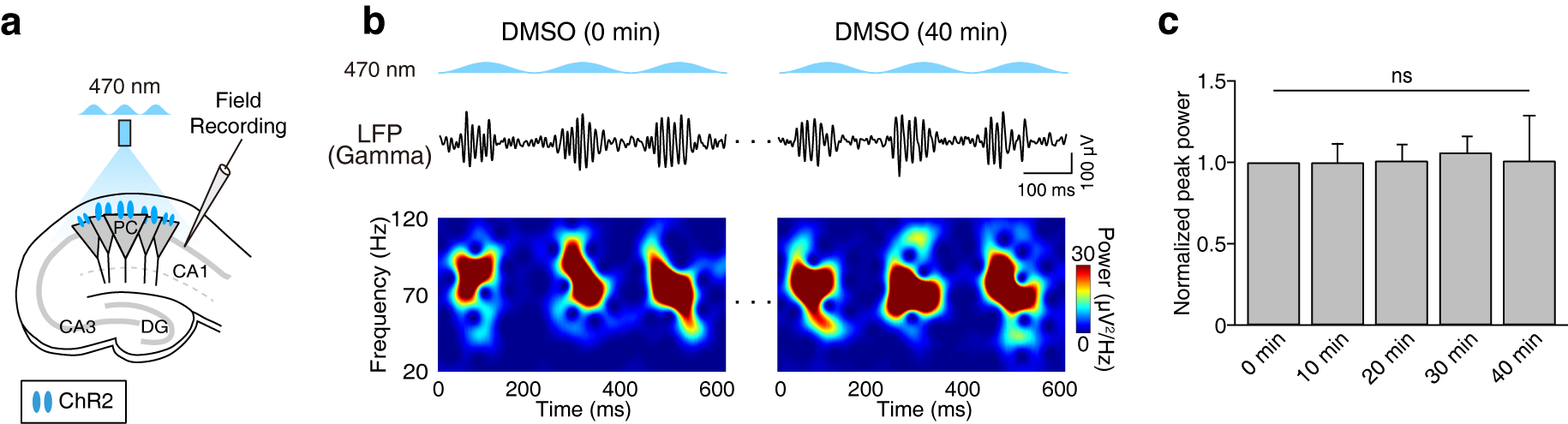
**

**Figure S2.** Stability of optogenetically-induced theta-nested gamma oscillations in hippocampal slices *in vitro*. **a** Experimental schematic showing sinusoidal (5 Hz) blue light (470 nm) stimulation of ChR2-expressing PC and field recording in CA1 area of DMSO-treated hippocampal slices *in vitro*. **b** Sinusoidal blue light stimulation induces theta-nested gamma oscillations as shown in the band-pass filtered LFP (top) and the corresponding spectrograms (bottom) in DMSO-treated slice. Three repetitions of 1 s-long sinusoidal blue light stimulations were delivered to the hippocampal slice every 10 min for 40 min. **c** Mean normalized peak power of blue light-induced gamma oscillations (*n* = 5) over 40 min. One-way repeated-measures ANOVA with *post-hoc* Tukey’s test (ns: not significant). Data are represented as mean ± SEM.
