# Supplementary material for: Optogenetic activation of parvalbumin and somatostatin interneurons selectively restores theta-nested gamma oscillations and oscillation-induced spike timing-dependent long-term potentiation impaired by amyloid β oligomers
Source: BMC Biol. 2020 Jan 15;18:7. doi: 10.1186/s12915-019-0732-7 (PMC6961381; doi:10.1186/s12915-019-0732-7)
Supplement: Supplementary file 3 — Additional file 3 : Figure S3. Impairment of optogenetically-induced theta-nested gamma oscillations in AβO1–42-treated hippocampal slice in vitro. [file 12915_2019_732_MOESM3_ESM.docx]

**Additional file 3**


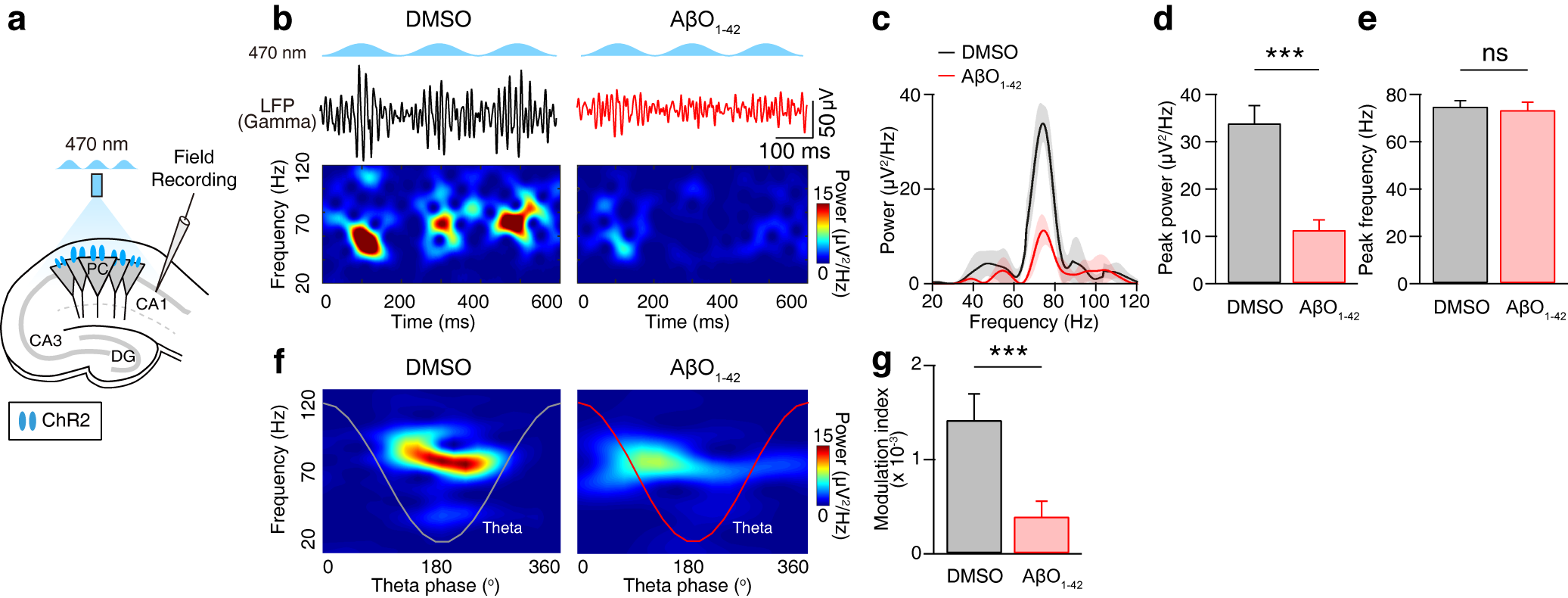


**Figure S3.** Impairment of optogenetically-induced theta-nested gamma oscillations in AβO_1-42_-treated hippocampal slice *in vitro*. **a** Experimental schematic showing sinusoidal (5 Hz) blue light (470 nm) stimulation of ChR2-expressing PC and field recording in CA1 area of hippocampal slices *in vitro*. **b** Sinusoidal blue light stimulation induces theta-nested gamma oscillations as shown in the band-pass filtered LFP (top) and the corresponding spectrograms (bottom) in DMSO-treated (left) and in a different slice treated with AβO_1-42_ before LFP recordings (right). **c-e** Representative PSD of gamma oscillations (**c**, shade indicates SEM), mean peak power (**d**), and mean peak frequency (**e**) of gamma oscillations in DMSO-treated (black, *n* = 10) and in a different AβO_1-42_-treated hippocampal slice (red, *n* = 9). **f, g** Representative comodulograms showing phase-amplitude coupling of gamma oscillations to theta cycle (**f**) and mean modulation index (**g**) in each condition. Unpaired Student’s *t-*test (****p* < 0.001, ns: not significant). Data are represented as mean ± SEM.
