# Supplementary material for: Optogenetic activation of parvalbumin and somatostatin interneurons selectively restores theta-nested gamma oscillations and oscillation-induced spike timing-dependent long-term potentiation impaired by amyloid β oligomers
Source: BMC Biol. 2020 Jan 15;18:7. doi: 10.1186/s12915-019-0732-7 (PMC6961381; doi:10.1186/s12915-019-0732-7)
Supplement: Supplementary file 4 — Additional file 4 : Figure S4. Intrinsic properties of SST and PV interneurons in DMSO- and AβO1–42-treated hippocampal slices in vitro. [file 12915_2019_732_MOESM4_ESM.docx]

**Additional file 4**


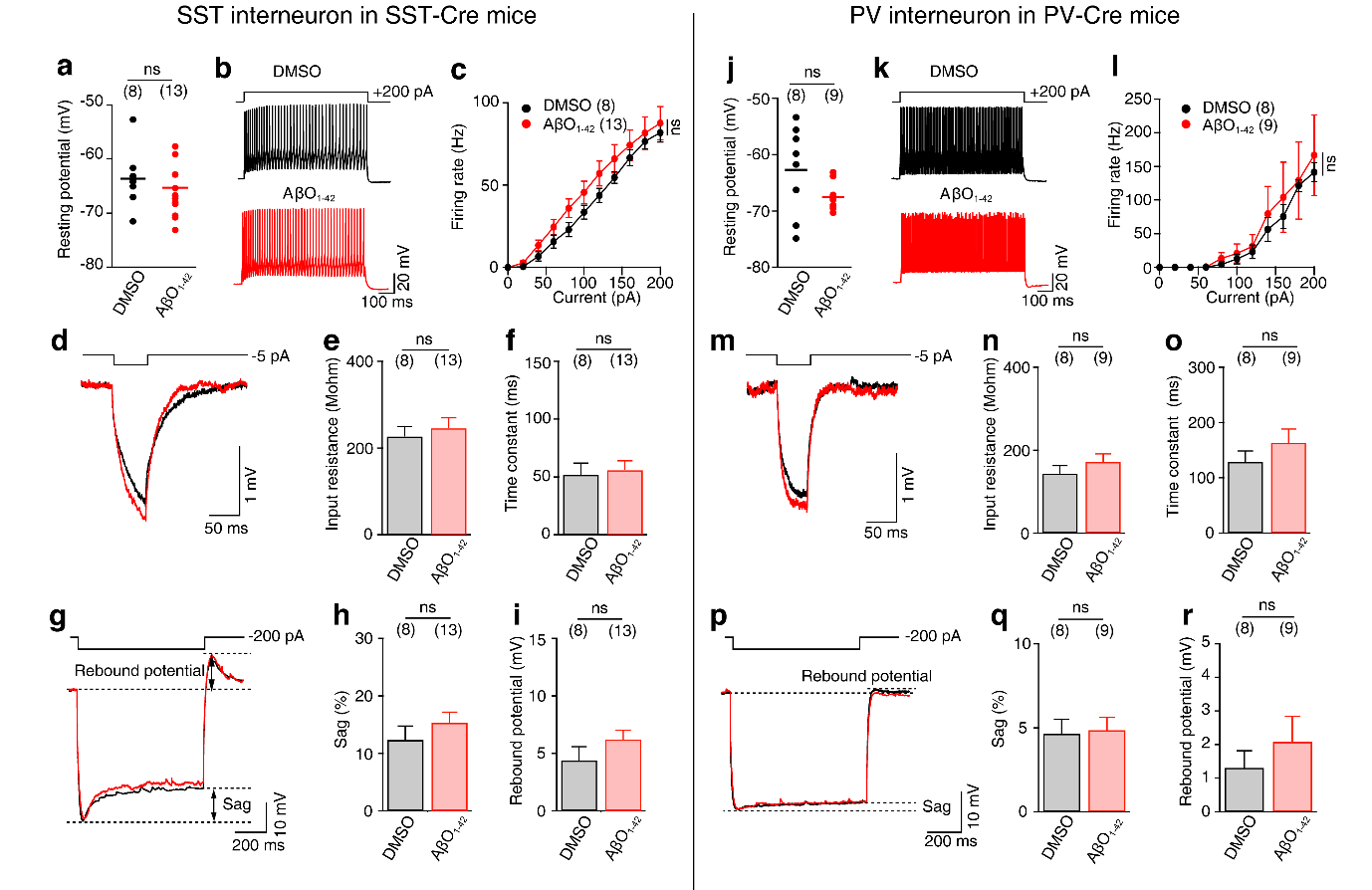


**Figure S4.** Intrinsic properties of SST and PV interneurons in DMSO- and AβO_1-42_-treated hippocampal slices *in vitro*. **a-c** Whole-cell current-clamp recordings in SST interneurons.  Resting membrane potential (**a**), voltage response to a depolarizing current step (**b**, +200 pA, 500 ms), and spike firing rate plotted as a function of depolarizing current steps in 20 pA increments (**c**) in DMSO-treated (black, *n* = 8) and AβO_1-42_-treated hippocampal slices (red, *n* = 13). **d-f** SST interneurons’ voltage response to hyperpolarizing current pulse (**d**, -5 pA, 50 ms) which was used to calculate input resistance (**e**) and time constant (**f**) by fitting an exponential curve in DMSO-treated (black, *n* = 8) and AβO_1-42_-treated hippocampal slices (red, *n* = 13). **g-i** SST interneurons’ voltage response to hyperpolarizing current step (**g**, -200 pA, 500 ms) which was used to calculate the sag (**h**) and rebound potential (**i**) in DMSO-treated (black, *n* = 8) and AβO_1-42_-treated hippocampal slices *in vitro* (red, *n* = 13). **j-r** Same as (**a-i**) but in whole-cell current-clamp recordings in PV interneurons in DMSO–treated (black, *n* = 8) and AβO_1-42_-treated hippocampal slices *in vitro* (red, *n* = 9). Unpaired Student’s *t-*test (**a, e, f, h, i, j, n, o, q** and **r**, ns: not significant) and two-way ANOVA with *post-hoc* Tukey’s test was performed (**c, l,** ns: not significant). Data are represented as mean ± SEM.
