# Supplementary material for: Optogenetic activation of parvalbumin and somatostatin interneurons selectively restores theta-nested gamma oscillations and oscillation-induced spike timing-dependent long-term potentiation impaired by amyloid β oligomers
Source: BMC Biol. 2020 Jan 15;18:7. doi: 10.1186/s12915-019-0732-7 (PMC6961381; doi:10.1186/s12915-019-0732-7)
Supplement: Supplementary file 6 — Additional file 6 : Figure S6. Optogenetic inactivation of Arch-expressing SST interneurons has no effect on the power of gamma oscillations. [file 12915_2019_732_MOESM6_ESM.docx]

**Additional file 6**

**
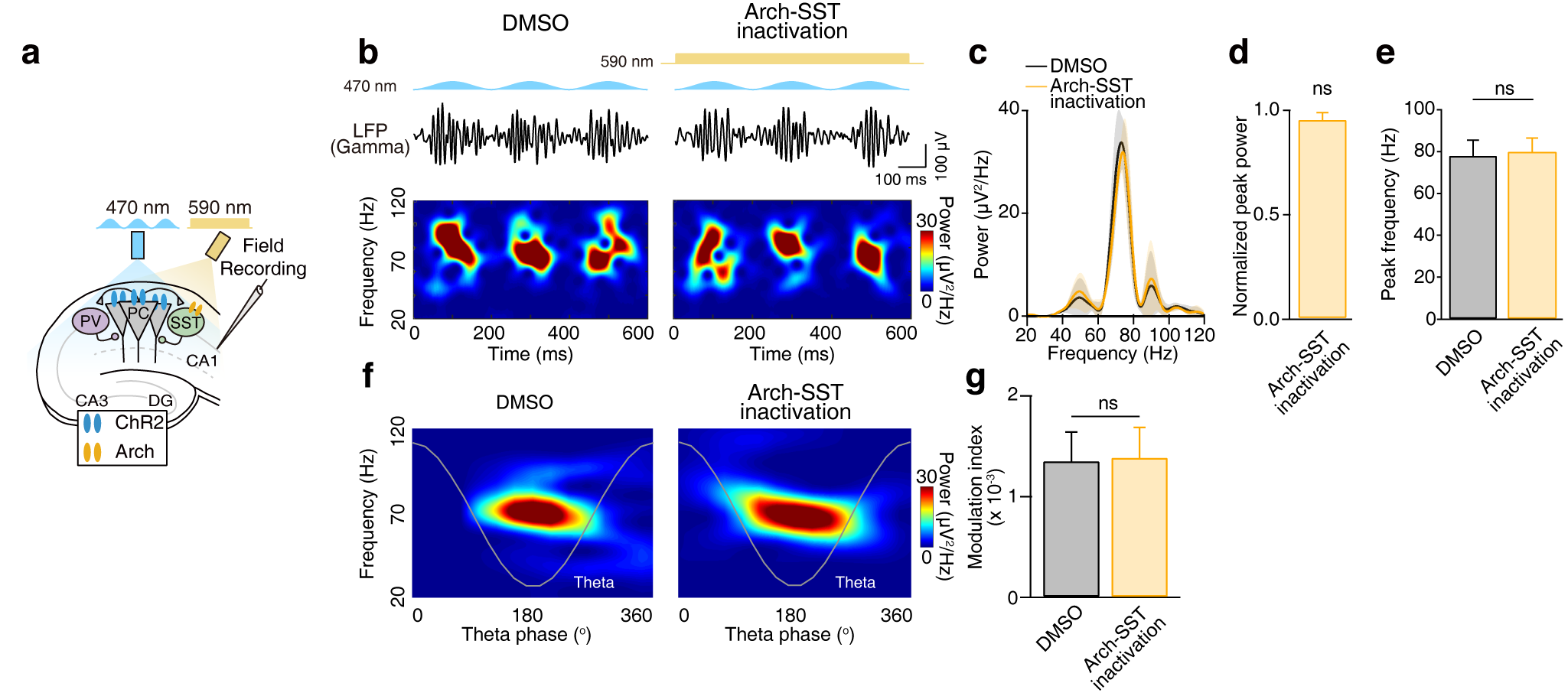
**

**Figure S6**. Optogenetic inactivation of Arch-expressing SST interneurons has no effect on the power of gamma oscillations. **a** Experimental schematic showing sinusoidal (5 Hz) blue light (470 nm) stimulation of ChR2-expressing PC, tonic yellow light (590 nm) stimulation of Arch-expressing SST interneuron (Arch-SST) and field recording in CA1 area of DMSO-treated hippocampal slices *in vitro*. **b** Sinusoidal blue light stimulation induces theta-nested gamma oscillations as shown in the band-pass filtered LFP (top) and the corresponding spectrograms (bottom) without (left) or with (right) tonic yellow light stimulation. **c-e** Representative PSD (**c**, shade indicates SEM), mean normalized peak power (**d**), and mean peak frequency (**e**) of the gamma oscillations without (black) and with tonic yellow light to inactivate Arch-SST (yellow) during blue light stimulation to induce gamma oscillations in DMSO-treated hippocampal slices (*n* = 6). **f, g** Representative comodulograms showing phase-amplitude coupling of gamma oscillations to theta cycle (**f**) and mean modulation index (**g**) in each condition. Paired Student’s *t-*test (ns: not significant). Data are represented as mean ± SEM.
