# Supplementary material for: Optogenetic activation of parvalbumin and somatostatin interneurons selectively restores theta-nested gamma oscillations and oscillation-induced spike timing-dependent long-term potentiation impaired by amyloid β oligomers
Source: BMC Biol. 2020 Jan 15;18:7. doi: 10.1186/s12915-019-0732-7 (PMC6961381; doi:10.1186/s12915-019-0732-7)
Supplement: Supplementary file 7 — Additional file 7 : Figure S7. Response of ChR2-expressing PC to different wavelengths of sinusoidal light stimuli. [file 12915_2019_732_MOESM7_ESM.docx]

**Additional file 7**


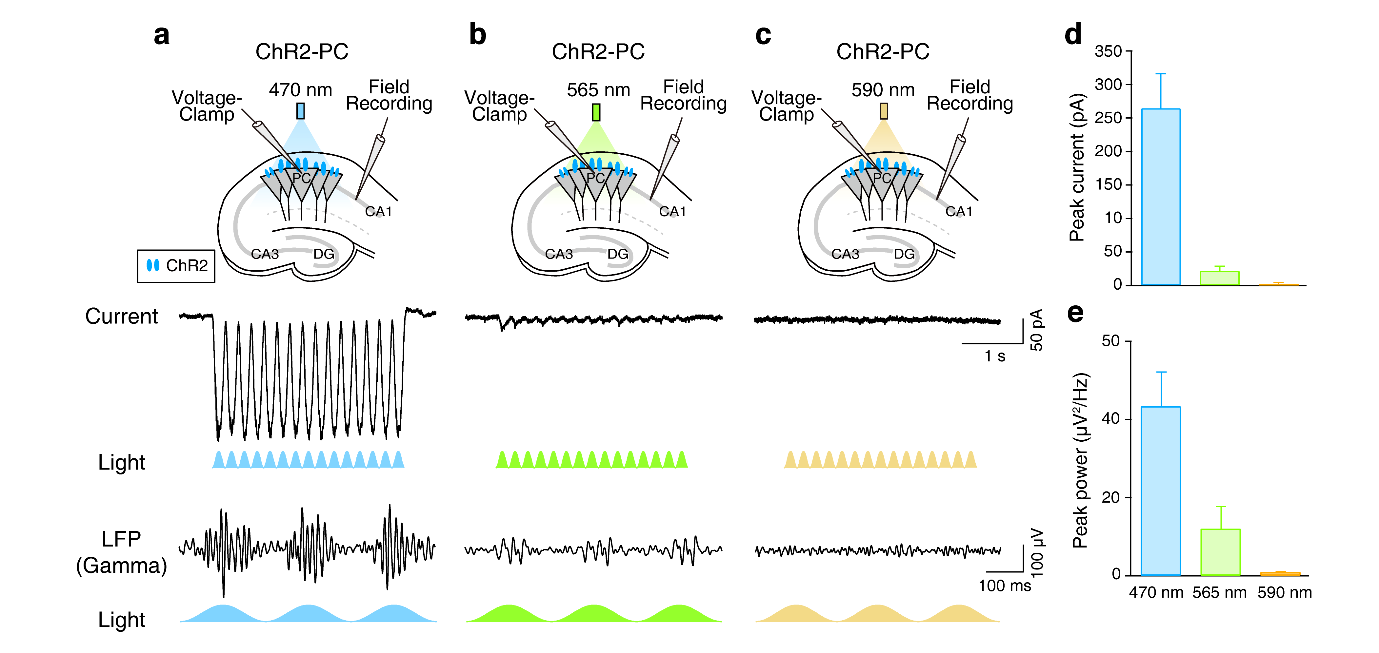


**Figure S7**. Response of ChR2-expressing PC to different wavelengths of sinusoidal light stimuli. **a-c** Experimental schematic showing whole-cell voltage-clamp recordings in ChR2-expressing PC (ChR2-PC) and field recording during sinusoidal (5 Hz) blue (**a**, 470 nm), green (**b**, 565 nm) and yellow (**c**, 590 nm) light stimulation (top). Representative traces of currents recorded from ChR2-PCs in response to each wavelength of light stimulation (middle). Representative traces of band-pass filtered LFP at gamma-frequency recorded in response to each wavelength of light stimulation (bottom). **d, e** Mean peak current (**d**, *n* = 6) and mean peak power of gamma oscillations (**e,** *n* = 4) in response to each wavelength of light stimulation. Data are represented as mean ± SEM.
