# Supplementary material for: Optogenetic activation of parvalbumin and somatostatin interneurons selectively restores theta-nested gamma oscillations and oscillation-induced spike timing-dependent long-term potentiation impaired by amyloid β oligomers
Source: BMC Biol. 2020 Jan 15;18:7. doi: 10.1186/s12915-019-0732-7 (PMC6961381; doi:10.1186/s12915-019-0732-7)
Supplement: Supplementary file 8 — Additional file 8 : Figure S8. Current response of Arch-expressing PV interneuron, Arch-expressing SST interneuron, and C1V1-expressing PV interneuron to 590 nm light stimulation. [file 12915_2019_732_MOESM8_ESM.docx]

**Additional file 8**


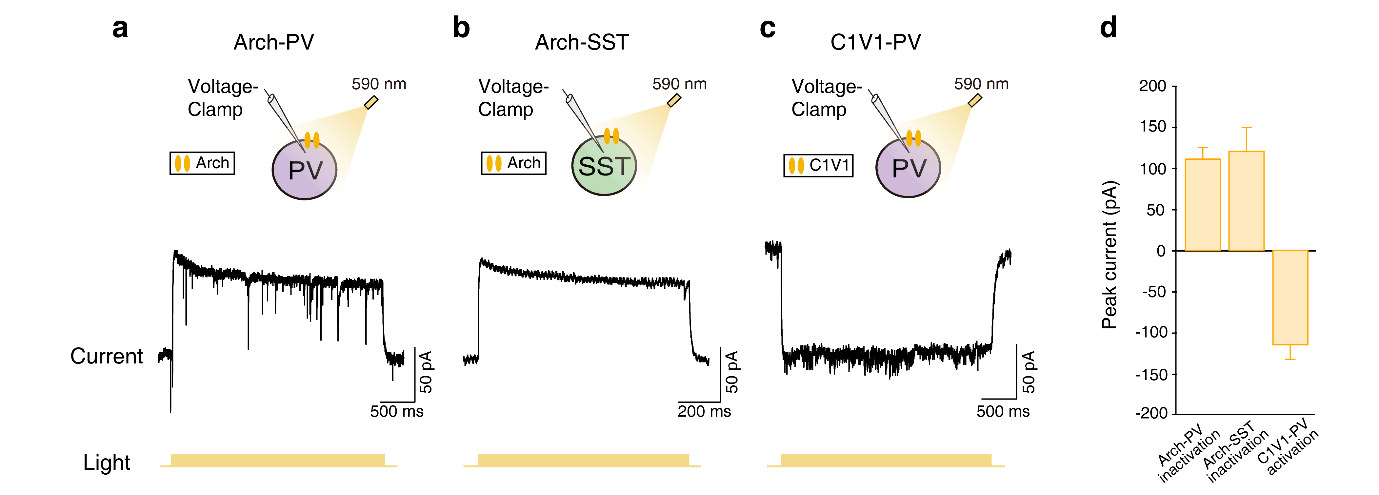


**Figure S8**. Current response of Arch-expressing PV interneuron, Arch-expressing SST interneuron, and C1V1-expressing PV interneuron to 590 nm light stimulation. **a-c** Experimental schematic showing whole-cell voltage-clamp recordings in Arch-expressing PV interneuron (**a**, Arch-PV), Arch-expressing SST interneuron (**b**, Arch-SST), and C1V1-expressing PV interneuron (**c**, C1V1-PV) during tonic yellow light stimulation (590 nm) (top). Representative traces of currents recorded in response to tonic yellow light stimulation in each cell type expressing different opsins (bottom). **d** Mean peak current from baseline (Arch-PV: *n* = 5, Arch-SST: *n* = 4, C1V1-PV: *n* = 5). Data are represented as mean ± SEM.
