# Supplementary material for: Optogenetic activation of parvalbumin and somatostatin interneurons selectively restores theta-nested gamma oscillations and oscillation-induced spike timing-dependent long-term potentiation impaired by amyloid β oligomers
Source: BMC Biol. 2020 Jan 15;18:7. doi: 10.1186/s12915-019-0732-7 (PMC6961381; doi:10.1186/s12915-019-0732-7)
Supplement: Supplementary file 9 — Additional file 9 : Figure S9. Stimulation of CA1 PC axons with a theta-nested gamma oscillation-like pattern entrains PV and SST interneurons at gamma frequency. [file 12915_2019_732_MOESM9_ESM.docx]

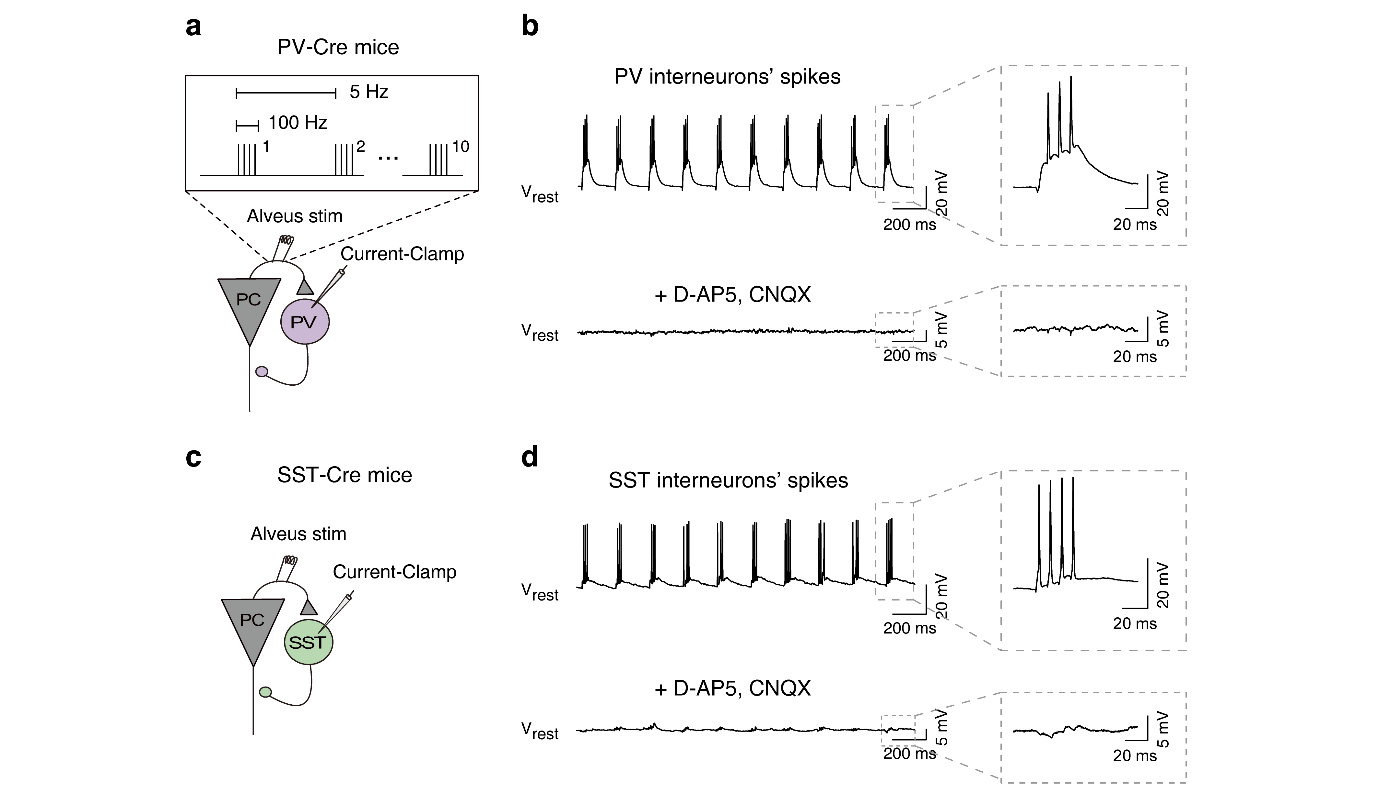
**Additional file 9**

**Figure S9.** Stimulation of CA1 PC axons with a theta-nested gamma oscillation-like pattern entrains PV and SST interneurons at gamma frequency. **a** Experimental schematic showing whole-cell current-clamp recordings in PV interneuron during alveus stimulation. Four stimuli were delivered at 100 Hz and repeated at 5 Hz to mimic CA1 PC spikes evoked during theta-nested gamma oscillations. **b** Spikes evoked by alveus stimulation (top) and the same experiment repeated in the presence of 50 μM D-AP5 and 20 μM CNQX to block NMDA and AMPA receptors (bottom) at resting membrane potential (V_rest_). **c, d** Same as (**a, b**) but with whole-cell current-clamp recordings in SST interneuron.
