# Supplementary material for: Optogenetic activation of parvalbumin and somatostatin interneurons selectively restores theta-nested gamma oscillations and oscillation-induced spike timing-dependent long-term potentiation impaired by amyloid β oligomers
Source: BMC Biol. 2020 Jan 15;18:7. doi: 10.1186/s12915-019-0732-7 (PMC6961381; doi:10.1186/s12915-019-0732-7)
Supplement: Supplementary file 10 — Additional file 10 : Figure S10. Experimental protocol for measuring SST interneuron-mediated disinhibition. [file 12915_2019_732_MOESM10_ESM.docx]

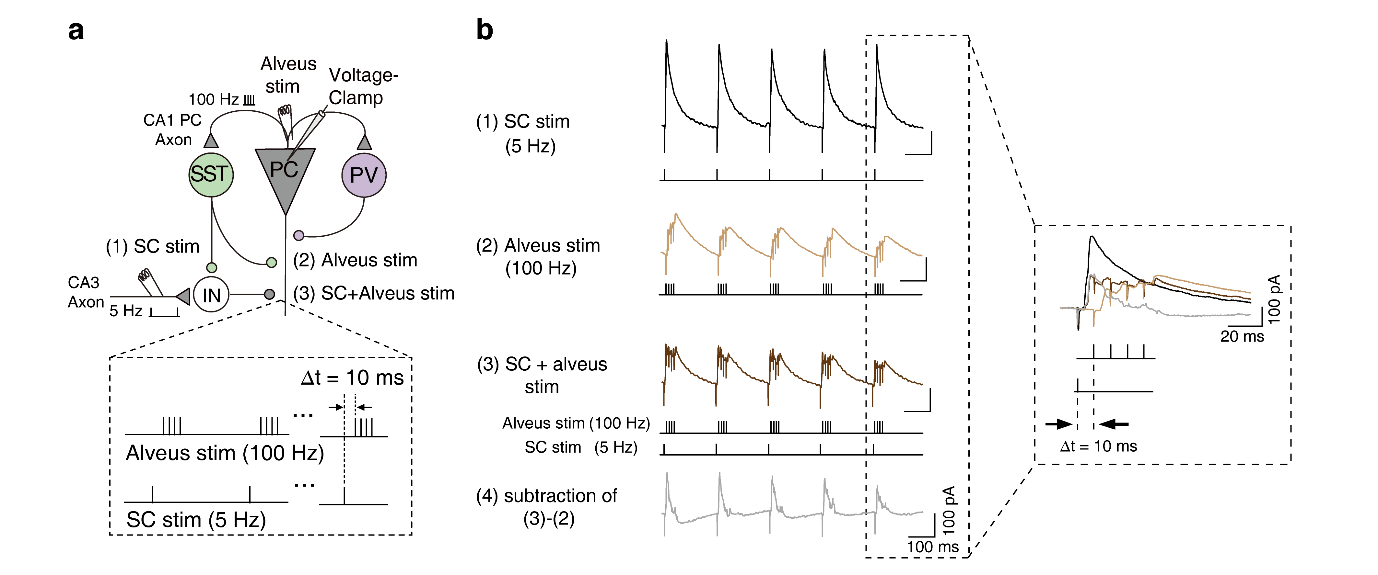
**Additional file 10**

**Figure S10.** Experimental protocol for measuring SST interneuron-mediated disinhibition. **a** Experimental schematic showing whole-cell voltage-clamp recordings in PC to record IPSC evoked by the stimulation of CA3 axon of Schaffer collateral (SC stim, 1), by the alveus stimulation of CA1 PC axons (Alveus stim, 2) and by the stimulation of both SC and alveus as shown in the dotted box (SC + Alveus stim, 3).  SC stimulation (1) is delivered once every 5 Hz. For alveus stimulation in (2), four stimuli at 100 Hz was delivered at 5 Hz to mimic theta-nested gamma oscillation-like spikes of PC. For SC + alveus stimulation (3), SC stimulation preceded the alveus stimulation (four pulses at 100 Hz) by 10 ms, which was repeated at 5 Hz to mimic spike timing-dependent long-term potentiation induction during theta-nested gamma oscillations. **b** Voltage responses for SC stimulation (black, 1), alveus stimulation (light brown, 2), SC + alveus stimulation (brown, 3) and subtraction of (3) from (2) to exclude IPSC that may have been evoked by PV and SST interneurons directly inhibiting PC by alveus stimulation (grey, 4) (left). Merged IPSC traces of each condition. All IPSCs were recorded at +10 mV (right).
