# Supplementary material for: Optogenetic activation of parvalbumin and somatostatin interneurons selectively restores theta-nested gamma oscillations and oscillation-induced spike timing-dependent long-term potentiation impaired by amyloid β oligomers
Source: BMC Biol. 2020 Jan 15;18:7. doi: 10.1186/s12915-019-0732-7 (PMC6961381; doi:10.1186/s12915-019-0732-7)
Supplement: Supplementary file 11 — Additional file 11 : Figure S11. Optical stimulation of ChR2-expressing SST interneurons restores AβO1–42-induced impairment of SST interneuron-mediated disinhibition. [file 12915_2019_732_MOESM11_ESM.docx]

**Additional file 11**


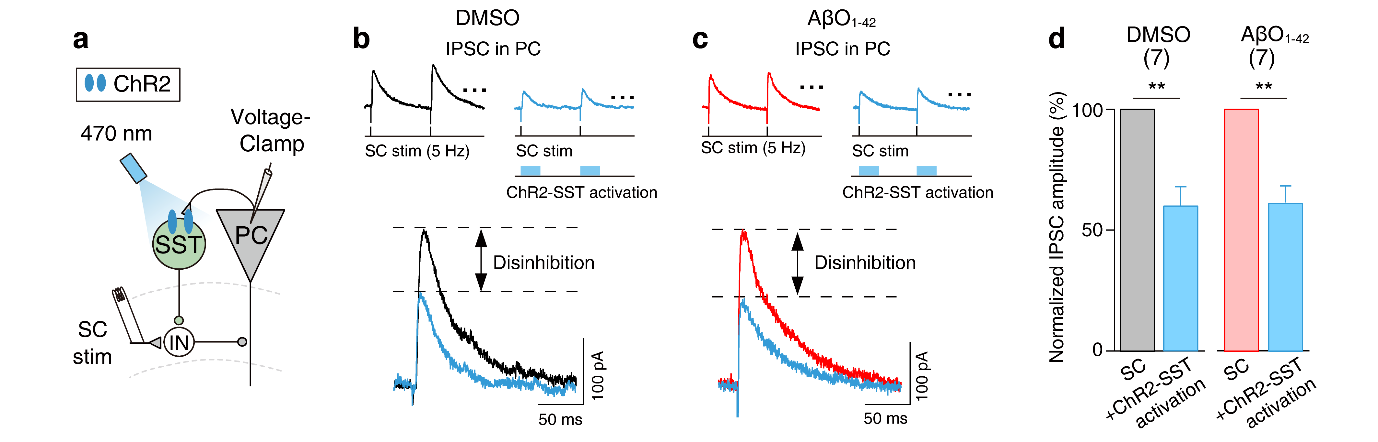


**Figure S11.** Optical stimulation of ChR2-expressing SST interneurons restores AβO_1-42_-induced impairment of SST interneuron-mediated disinhibition. **a** Experimental setup for voltage-clamp recordings of IPSCs in CA1 PCs during direct optical stimulation of ChR2-expressing SST interneuron (ChR2-SST) paired with Schaffer collateral (SC) stimulation. **b** IPSC evoked by SC stimulation alone (black) and pairing of SC stimulation with 50 ms blue light (ChR2-SST activation) (blue) in DMSO-treated hippocampal slices (top) and the two IPSCs superimposed together (bottom). **c** Same experiment in (**b**) but repeated in AβO_1-42_-treated hippocampal slices. **d** Normalized IPSC amplitudes evoked by the pairing of SC stimulation and 50 ms blue light (ChR2-SST activation, blue) by IPSC evoked by SC stimulation alone in DMSO-treated hippocampal slices (black, *n* = 7) and in AβO_1-42_-treated hippocampal slices (red, *n* = 7). Paired Student’s *t-*test between SC and +ChR2-SST activation in (**d**, ** *p* < 0.01). Data are represented as mean ± SEM.
