# Supplementary material for: Optogenetic activation of parvalbumin and somatostatin interneurons selectively restores theta-nested gamma oscillations and oscillation-induced spike timing-dependent long-term potentiation impaired by amyloid β oligomers
Source: BMC Biol. 2020 Jan 15;18:7. doi: 10.1186/s12915-019-0732-7 (PMC6961381; doi:10.1186/s12915-019-0732-7)
Supplement: Supplementary file 12 — Additional file 12 : Figure S12. The effect of SST interneuron activation on spike firing rates and spike phases of CA1 PC and PV interneurons during theta-nested gamma oscillations in silico. [file 12915_2019_732_MOESM12_ESM.docx]

**Additional file 12**


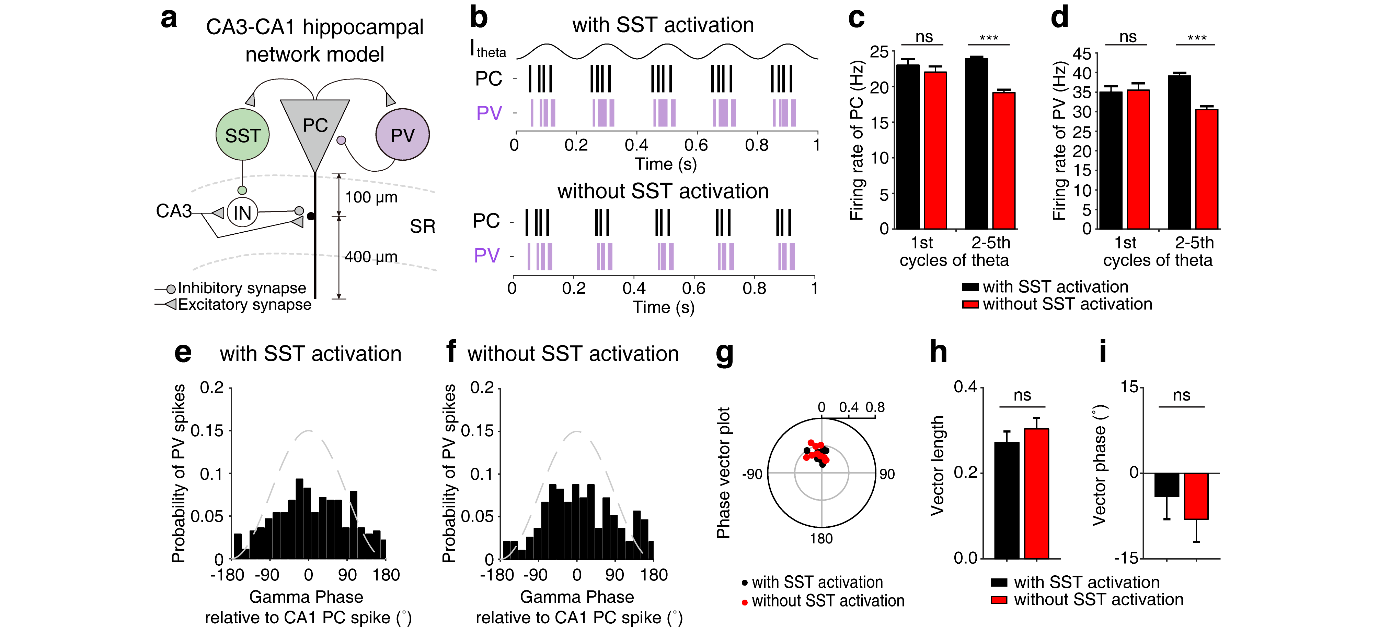


**Figure S12.** The effect of SST interneuron activation on spike firing rates and spike phases of CA1 PC and PV interneurons during theta-nested gamma oscillations *in silico*. **a** Schematic diagram of CA3-CA1 hippocampal network model. **b** The spike raster plot of PC and PV interneuron models with SST activation (top) and without SST activation (bottom) during theta oscillations current (I_theta_, 5 Hz). **c, d** Firing rates of CA1 PC (**c**) and PV interneuron (**d**) with SST activation (black) and without SST activation (red) in the network model during the first theta cycle and during the successive theta cycles. **e, f** Phase histogram of PV interneuron spike phases relative to CA1 PC-generated gamma-frequency spikes with SST activation (**e**) or without SST activation (**f**). **g-i** Vector phase and length of PV interneuron spike phases relative to CA1 PC-generated gamma-frequency spikes plotted in polar plot (**g**), mean vector length (**h**), and circular mean vector phase of PV interneuron model’s spikes (**i**) with SST activation (black) and without SST activation (red) in the network model. Student’s *t*-test in (**c, d, h,** *** *p* < 0.001, ns: not significant) and Watson-Williams test in (**i**, ns: not significant). Data are represented as mean ± SEM (n = 10 simulations).
