# Supplementary material for: Optogenetic activation of parvalbumin and somatostatin interneurons selectively restores theta-nested gamma oscillations and oscillation-induced spike timing-dependent long-term potentiation impaired by amyloid β oligomers
Source: BMC Biol. 2020 Jan 15;18:7. doi: 10.1186/s12915-019-0732-7 (PMC6961381; doi:10.1186/s12915-019-0732-7)
Supplement: Supplementary file 13 — Additional file 13 : Table S1. Parameters of CA1 PC, PV, SST and IN models. [file 12915_2019_732_MOESM13_ESM.docx]

**Additional file 13**

**Table S1.** Parameters of CA1 PC, PV, SST and IN models.

| Parameters | PC  soma | PC  dendrite | PC  spine | PV  model | SST  model | IN  Model |
| --- | --- | --- | --- | --- | --- | --- |
| Diameter (μm) | 7.14 | 1.5 | 0.35 | 16 | 40 | 46 |
| Length (μm) | 7.14 | 585 | 0.35 | 16 | 40 | 46 |
| Resting Potential (mV) | -60 | | | -65 | -65 | -65 |
| Capacitance (μF/cm^2^) | 1 | | | 1 | 1 | 1 |
| Axial Resistance (Ω cm) | 200 | | | 100 | 100 | 100 |
| g_Leak_ (S/cm^2^) | 0.0000375 | | | 0.00106 | 0.00008 | 0.000032 |
| g_Na_ (S/cm^2^) | 0.025 | 0.025 | 0.007 | 0.146 | 0.05 | 0.011 |
| g_KDR_ (S/cm^2^) | 0.0031 | 0.0031 | 0.000867 | 0.027 | 0.009 | 0.01 |
| g_KA_ (S/cm^2^) | 0.042 | 0.042 | 0.012 | 0.0047 | 0.0013 | 0.0034 |
| g_CaL_ (S/cm^2^) | 0.00015 | 0.00015 | 0.014 |  |  |  |
| g_AHP_ (S/cm^2^) | 0.0005 |  |  |  |  |  |
| g_h_ (S/cm^2^) | 0.00002 |  |  |  |  |  |
| g_KM_ (S/cm^2^) | 0.003 |  |  |  |  |  |
