# Supplementary material for: Optogenetic activation of parvalbumin and somatostatin interneurons selectively restores theta-nested gamma oscillations and oscillation-induced spike timing-dependent long-term potentiation impaired by amyloid β oligomers
Source: BMC Biol. 2020 Jan 15;18:7. doi: 10.1186/s12915-019-0732-7 (PMC6961381; doi:10.1186/s12915-019-0732-7)
Supplement: Supplementary file 14 — Additional file 14 : Table S2. Parameters of the deterministic Ca2+-dependent STDP model. [file 12915_2019_732_MOESM14_ESM.docx]

**Additional file 14**

**Table S2.** Parameters of the deterministic Ca^2+^-dependent STDP model.

| Parameter | Value | Parameter | Value |
| --- | --- | --- | --- |
| pHC (μM) | 4 | pHN | 4 |
| aHC (μM) | 0.6 | aHN | 3 |
| θ_v_ (μM) | 2 | σ_v_ | -0.05 |
| θ_d_ | 2.5 | σ_d_ | -0.01 |
| θ_b_ | 0.55 | σ_b_ | -0.02 |
| τ_p_ (ms) | 500 | k_p_ | -0.015 |
| τ_a_ (ms) | 5 | k_d_ | -0.0015 |
| τ_v_ (ms) | 10 | α_v_ | 1.0 |
| τ_d_ (ms) | 250 | α_d_ | 1.0 |
| τ_b_ (ms) | 40 | α_b_ | 5.0 |
| c_p_ | 5 | c_d_ | 4 |
| τ_w_ (ms) | 500 | p | 0.2 |
| α_w_ | 0.8 | d | 0.01 |
| β_w_ | 0.6` |  |  |
